# LKB1 deficiency upregulates RELM-α to drive airway goblet cell metaplasia

**DOI:** 10.1007/s00018-021-04044-w

**Published:** 2021-12-18

**Authors:** Yu Li, Qiuyang Zhang, Li Li, De Hao, Peiyong Cheng, Kuan Li, Xue Li, Jianhai Wang, Qi Wang, Zhongchao Du, Hongbin Ji, Huaiyong Chen

**Affiliations:** 1 Department of Basic Medicine, Haihe Hospital, Tianjin University, Tianjin, 300350 China; 2 Key Research Laboratory for Infectious Disease Prevention for State Administration of Traditional Chinese Medicine, Tianjin Institute of Respiratory Diseases, Tianjin, China; 3 Department of Basic Medicine, Haihe Clinical School, Tianjin Medical University, Tianjin, China; 4 Tianjin Key Laboratory of Lung Regenerative Medicine, Tianjin, China; 5 Department of Respiratory Medicine, Haihe Clinical School, Tianjin Medical University, Tianjin, China; 6 State Key Laboratory of Cell Biology, CAS Center for Excellence in Molecular Cell Science, Shanghai Institute of Biochemistry and Cell Biology, Chinese Academy of Sciences, University of Chinese Academy of Sciences, Shanghai, China

**Keywords:** Cystic fibrosis, Asthma, Lung stem and progenitor cells, Crosstalk, Cell fate, Polarization

## Abstract

Targeting airway goblet cell metaplasia is a novel strategy that can potentially reduce the chronic obstructive pulmonary disease (COPD) symptoms. Tumor suppressor liver kinase B1 (LKB1) is an important regulator of the proliferation and differentiation of stem/progenitor cells. In this study, we report that LKB1 expression was downregulated in the lungs of patients with COPD and in those of cigarette smoke-exposed mice. *Nkx2.1*^*Cre*^*; Lkb1*^*f/f*^ mice with conditional loss of *Lkb1* in mouse lung epithelium displayed airway mucus hypersecretion and pulmonary macrophage infiltration. Single-cell transcriptomic analysis of the lung tissues from *Nkx2.1*^*Cre*^*; Lkb1*^*f/f*^ mice further revealed that airway goblet cell differentiation was altered in the absence of LKB1. An organoid culture study demonstrated that Lkb1 deficiency in mouse airway (club) progenitor cells promoted the expression of FIZZ1/RELM-α, which drove airway goblet cell differentiation and pulmonary macrophage recruitment. Additionally, monocyte-derived macrophages in the lungs of *Nkx2.1*^*Cre*^*; Lkb1*^*f/f*^ mice exhibited an alternatively activated M2 phenotype, while expressing RELM-α, which subsequently aggravated airway goblet cell metaplasia. Our findings suggest that the LKB1-mediated crosstalk between airway progenitor cells and macrophages regulates airway goblet cell metaplasia. Moreover, our data suggest that LKB1 agonists might serve as a potential therapeutic option to treat respiratory disorders associated with goblet cell metaplasia.

**Supplementary Information:**

The online version contains supplementary material available at 10.1007/s00018-021-04044-w.

## Introduction

Airway mucus is an important part of the fundamental host defense system that enables the clearing of inhaled particles by trapping pathogens to preserve airway sterility and homeostasis. However, goblet cell metaplasia-associated excessive airway mucus is one of the most common symptoms of several lung diseases, including asthma, cystic fibrosis, and chronic obstructive pulmonary disease (COPD), leading to severe clinical outcomes in patients [1–3]. Hence, goblet cell fate determination is meticulously regulated during both lung development and postnatal lung epithelial homeostasis, as maintaining an adequate number of mucus-producing goblet cells is not only important for securing a basal mucus secretion in the airways, but also for preventing the occurrence of mucus plugging in the bronchioles [4, 5]. Goblet cells are mainly derived from secretoglobin family 1A member 1 (Scgb1a1, also called as CCSP)-expressing club (previously called Clara) cells and are believed to be tissue-resident facultative epithelial progenitors under the control of physiological or injury-associated signals [6, 7]. The transcription factor SAM-pointed domain containing Ets transcription factor (SPDEF) activates forkhead box A3 (Foxa3) to induce the differentiation of club cells into goblet cells [8]. Foxp1/4 restricts goblet cell differentiation during lung development and regeneration by regulating the anterior gradient 2 (*Agr2*) [4]. Notch signaling interacts with other pathways through downstream elements, including epidermal growth factor receptor, to direct goblet cell differentiation in the airway epithelium [9–12]. The club cell-specific activation of β-catenin at the later stages of lung development leads to goblet cell metaplasia, which is associated with an increased secretion of mucins [13]. The epithelial-specific deletion of receptor-like tyrosine kinase (*Ryk*) gradually increases the number of goblet cells during lung development, while the club cell-specific deletion of *Ryk* in adult stages also drives goblet cell metaplasia and mucus hypersecretion during regeneration [5]. In addition, external interleukin 33 (IL-33) induces goblet cell differentiation in the intestine and lungs through IL-13, which is usually produced by type-2 innate lymphoid cells [14, 15]. The number and activity of goblet cells is induced by various inflammatory stimuli. However, the regulation of goblet cell differentiation by inflammatory cells remains largely unexplored.

Resistin-like molecule-α (RELM-α, also known as HIMF or FIZZ1), which is a protein produced and secreted by alternatively activated macrophages, epithelial cells, eosinophils, and microglial cells, is highly induced by IL-13 [16–18]. RELM-α expression is significantly elevated in the lungs during airway inflammation [19, 20]. The intranasal *Alternaria* administration-induced expression of RELM-α results in airway eosinophilia, epithelial thickening, and fibrosis [21]. In addition, RELM-α promotes the production of monocyte chemotactic protein-1 (MCP-1) by lung endothelial cells [22]. The transgenic expression of RELM-α in club cells has been shown to increase the abundance of macrophages in bronchoalveolar lavage during silica-induced inflammation [23]. However, the crosstalk between epithelial cells and macrophages in goblet cell fate specification remains largely unknown.

Liver kinase B1 (LKB1), which is encoded by serine/threonine kinase 11 (*Stk11*), is a tumor suppressor and its inactivation has been demonstrated to drive the development of lung cancer, melanoma, prostate cancer, and cervical cancer [24–26]. The loss of LKB1 results in altered energy metabolism, impaired integrity, and disrupted cell polarity in mammalian epithelial tissues [27–30]. LKB1 deficiency has been demonstrated to impair ciliated cell differentiation in the fallopian tube and lung epithelium [31, 32]. In this study, we employed single-cell RNA sequencing (scRNA-seq) analysis, organoid cultures, transgenic tools, and immunostaining techniques to investigate the role of LKB1 in lung goblet cell fate specification. We found that LKB1 was expressed in club cells, and that adult mice with LKB1 deleted in the embryonic lung epithelium displayed goblet cell metaplasia in the lungs. Furthermore, silencing the epithelial *Lkb1* increased the expression of RELM-α, which drives goblet cell differentiation, macrophage accumulation, and activation in the lungs. Thus, macrophage-derived RELM-α contributes to the further development of airway goblet cell metaplasia. This study provides a novel insight into the crosstalk between macrophages and club cells in goblet cell lineage commitment in adult airways.

## Materials and methods

### Patient tissue samples

The COPD human lung specimens were bronchoscopically obtained from patients with COPD. The normal human lung specimens were obtained from the para-carcinoma tissue of patients with lung cancer during operation. The study was performed in accordance with the 1964 Declaration of Helsinki. All the human lung specimens were obtained from the Research Biorepository under the approval of the Research Ethics Committee of Haihe Hospital, Tianjin University (2021HHKT-018).

### Mice

NK2 homeobox 1 (*Nkx2.1)-Cre* transgenic mice were obtained from Jackson Laboratory (Bar Harbor, ME, USA). *Lkb1*^f/f^ mice were generously gifted by Dr. Ronald DePinho (Boston, MA, USA). *Scgb1a1*^*CreER*^ mice were obtained from The Jackson Laboratory (Bar Harbor, ME, USA). C57BL/6J mice were purchased from Beijing Huafukang Bioscience Co., Ltd. (Beijing, People’s Republic of China). To induce Lkb1 knockdown in the mouse lung epithelium, *Nkx2.1*^*Cre*^*; Lkb1*^f/f^ mice were established by crossing *Nkx2.1-Cre* mice with *Lkb1*^f/f^ mice. *Scgb1a1*^*CreER*^*; Lkb1*^*f/f*^ mice were established by crossing *Scgb1a1*^*CreER*^ mice with *Lkb1*^f/f^ mice. Tamoxifen was injected intraperitoneally at 200 mg/kg every other day for three times to induce the deletion of Lkb1 in club cells. Mice were maintained in a specific pathogen-free facility at the Tianjin University Haihe Hospital. All animal studies were approved by the Animal Care and Use Committee of the Tianjin University Haihe Hospital.

### Cell culture

The mouse lung fibroblast cell line MLg 2908 (MLg) was purchased from the American Type Culture Collection (Manassas, VA, USA). MLg cells were cultured in DMEM supplemented with 10% FBS, 100 IU/mL penicillin, and 100 μg/mL streptomycin in a humidified incubator at 37 °C and 5% CO_2_.

### Fluorescence-activated cell sorting

Mouse lungs were dissociated via elastase digestion, as described previously [6]. Briefly, the lungs were perfused with 4 U/mL elastase (Worthington Biochemical Corporation, Lakewood, NJ) and finely minced in a dish containing DNase I (Sigma-Aldrich, St. Louis, MO). The single-cell suspensions prepared for fluorescence-activated cell sorting (FACS) were then passed through a 100 μm strainer (Falcon; BD Biosciences, San Jose, CA) and collected in a 50 mL tube, and RBC lysis buffer (eBioscience, San Diego, CA, USA) was added to the tube. Cells were resuspended in Hanks’ balanced salt solution supplemented with 2% fetal bovine serum (FBS), 10 mM HEPES, 100 mg/mL streptomycin, 100 IU/mL penicillin, and 0.1 mM EDTA. Flow cytometry was performed to sort the club cells from the mouse lung tissues using primary antibodies, including anti-CD31-biotin, anti-CD34-biotin, anti-CD45-biotin, anti-CD24-phycoerythrin (PE), anti-EpCAM-PE-Cyanine 7 (PE-Cy7), Sca-1-allophycocyanin (APC), and the secondary antibody streptavidin APC-eFluor 780. The sorting of macrophages from the mouse lung tissues was performed using APC-Cy7-conjugated anti-mouse CD11b antibody. The dead cells were stained with 7-amino-actinomycin D (7-AAD). All antibodies were obtained from eBioscience (San Diego, CA, USA). Fluorescence-activated cell sorting (FACS) was performed using a FACS Aria III sorter (BD Biosciences, San Jose, CA, USA).

### 3D organoid culture

As described previously [6], FACS-sorted mouse EpCAM^+^ CD24^+^ Sca-1^+^ airway club cells (3000 cells per well) were co-cultured with MLg lung fibroblast cells in Matrigel. The cell mixture was seeded in the inserts, which were then placed into the wells of a 24-well plate containing basal DMEM culture medium (Cellgro, Manassas, VA) supplemented with 10 μM SB43142. Organoid cultures were maintained at 37 °C in a 5% CO_2_ incubator, and the culture medium was replaced every other day. Colonies were visualized and counted on day 10 after seeding. In some experiments, club cells were co-cultured with CD11b^+^ macrophages (1500 cells per well) in the organoids. Organoid cultures were embedded in paraffin for immunofluorescent staining or lysed using TRIzol reagent for gene expression analysis.

### Quantitative RT-PCR

The total RNA was extracted from the organoids or lung tissues using TRIzol reagent (Invitrogen) and then reverse-transcribed using M-MLV Reverse Transcriptase (Promega Corporation, Madison, WI, USA). qPCR was conducted using SYBR Select Master Mix (Applied Biosystems, Thermo Fisher Scientific Corporation, Foster City, CA, USA). The relative expression levels of the target genes were normalized to that of the housekeeping gene, *Actb*, for the same sample. The primer sequences designed for this study are as follows: *Muc5AC*-F, 5′-TGACTCAATCTGCGTGCCTT-3′; *Muc5Ac*-R, 5′-AGGCCTTCTTTTGGCAGGTT-3′; *CLCA3*-F, 5′-GGCATCGTCATCGCCATAG-3′; *CLCA3*-R, 5′-CACCATGTCCTTTATGTGTTGAATG-3′; *Foxa3*-F, 5′-CTTGGTGGAGGTTGGGTGAG-3′; *Foxa3*-R, 5′-ACAGGCAGTATTCCCAAGCC-3′; *Spdef*-F, 5′-GACTGTGGAATTCCTGGGGG-3′; *Spdef*-R, 5′-ATTGTGGCAGGAGCAGAGAC-3′; *β-actin*-F, 5′-GGCCAACCGTGAAAAGATGA-3′; *β-actin*-R, 5′-CAGCCTGGATGGCTACGTACA-3′.

### Hematoxylin and eosin staining

The rehydrated sections were stained with hematoxylin (ZSGB-BIO, Beijing, People’s Republic of China) for 3 min, rinsed in water for 10 min, and dehydrated in 85% and 95% alcohol for 2 min each. They were subsequently stained with an eosin solution (ZSQB-BIO) for 30 s, followed by dehydration in 95% and 100% alcohol for 3 min each, and cleared in xylene twice for 5 min each. The slides were then mounted using a neutral balsam.

### Alcian blue staining

Alcian blue staining was performed to identify the goblet cells. The rehydrated sections were stained with 1% Alcian blue (Poly Scientific R&D Corp., Bay Shore, NY, USA) for 10 min and rinsed in running water for 10 min. They were subsequently stained with an eosin solution (ZSQB-BIO) for 30 s, dehydrated, and mounted using a neutral balsam.

### Immunofluorescent microscopy

The paraffin-embedded sections (5 μm) of mouse lungs or organoids were subjected to antigen retrieval in citric acid (10 mM, pH 6), blocked with 5% bovine serum albumin in 0.2% Triton-X/PBS, and then stained with the primary antibodies at the following dilutions: rabbit anti-ClCa3 (1:200, Invitrogen), mouse anti-CYP2F2 (1:100, Santa Cruz Biotechnology, Inc., Dallas, TX, USA), rabbit anti-YM1/2 (1:200, Abcam, Cambridge, UK), and rabbit anti-RELM-α (1:200, Abcam). The sections were incubated with Alexa fluorophore-conjugated secondary antibodies (1:200, Invitrogen) at a 1:200 dilution. Then, they were mounted using Fluoromount G containing DAPI and imaged using an IX73 inverted fluorescence microscope (Olympus, Tokyo, Japan) or a laser confocal microscope (Leica TCS SP8). We also used a confocal microscope (DMi8 CS Bino, Leica) to collect fluorescent images using a 10 × objective (Leica, HC PL APO 10 × /0.45), a HIVIS scan optics module with rotation, and an electron-multiplying charge-coupled device camera (Evolve 512; Photometrics). We added scale bars, and processed the images using LAS X (Leica) and Photoshop (Adobe). For the quantification of images, three or more regions were analyzed in each section.

### Transcriptomic dataset retrieval

The transcriptomic data of the lung tissues of patients with COPD (GSE106986) and with airway epithelial brushings of asthma (GSE67472), the lung tissues of cigarette smoke-exposed mice (GSE125521), the lung tissues of house dust mite-exposed mice (GSE71822), and the single-cell RNA-seq data of the airway epithelial cells from patients with cystic fibrosis (GSE150674) were obtained from the series matrix files downloaded from the NCBI Gene Expression Omnibus (GEO) database. These transcriptomic data were normalized based on the Counts per Million function and/or the log2 function before the analysis of LKB1 expression.

### Single-cell RNA-seq and data analysis

The total lung cells isolated from three *Lkb1*^*f/f*^ or *Nkx2.1*^*Cre*^*; Lkb1*^*f/f*^ mice were pooled and processed to prepare scRNA-seq libraries using Chromium Single Cell 3′ Reagent v3 Kits (10 × Genomics, Pleasanton, CA, USA). Single-cell suspensions were loaded onto a chromium single-cell controller instrument (10XGenomics, Pleasanton, CA, USA) to generate single-cell gel beads in emulsions (GEMs). After the generation of the GEMs, reverse transcription, cDNA PCR amplification, and library preparation were performed according to the 10 × Genomics 3′ gene expression protocol. These libraries were pooled and sequenced using the HiSeq NovaSeq platform (Illumina, San Diego, CA, USA) in the 150 bp paired-end mode. Single-cell RNA-seq data preprocessing scRNA-seq analyses resulted in demultiplexed cellular barcodes and the reads were mapped to the mouse genome (version mm10) using the Cell Ranger pipeline (version 3.0.0) provided by 10 × Genomics. Seurat (version 3.5.0) was used for subsequent analysis [33]. To remove the low-quality cells and multiple captures, cells were removed if they expressed fewer than 200 unique genes or 500 UMI and more than 5000 unique genes or 50,000 UMI. In addition to these threshold values, we further removed the cells with more than 20% mitochondrial-derived UMI counts. Then, the QC criteria were applied. To reduce the dimensionality of the scRNA-seq dataset, principal component analysis was performed using the RunPCA function in Seurat. For tSNE projection and clustering analysis, the cells were clustered considering a graph-based clustering approach and were visualized in 2D using tSNE. The marker genes of each cluster were identified using the FindAllMarkers function (test. use = bimod) in Seurat. We used the FindMarkers function in Seurat to identify the differentially expressed genes (DEGs) [33]. A *p* < 0.05 and a |log2foldchange|> 0.58 were set as the threshold values for a significant differential expression. Gene ontology enrichment analyses of the DEGs were performed using the R software based on a hypergeometric distribution.

### Pseudotime analysis

Pseudotime analysis was performed on club and goblet cells using the Monocle2 R package (version 2.18.0). The club and goblet cells on the pseudotime trajectory were derived from *Nkx2.1*^*Cre*^*; Lkb1*^*f/f*^ and *Lkb1*^*f/f*^ mice.

### Gene set variation analysis (GSVA)

We performed pathway analyses of the Kyoto Encyclopedia of Genes and Genomes (KEGG) subset of canonical pathways described in the C2 curated gene sets of the molecular signature database (MSigDBwith) of the GSEABase package (version 1.52.1). The GSVA package (version 1.38.2) was used to evaluate the pathway activity estimates.

### Transcription factor (TF) regulatory network analysis

We used single-cell regulatory network inference and clustering (SCENIC; version 1.2.4) to perform TF regulatory network analysis. Using RcisTarget (version 1.10.0), mmp9 TFs were downloaded and used as a reference. The DEGs in the lung epithelial cells between *Nkx2.1*^*Cre*^*; Lkb1*^*f/f*^ and *Lkb1*^*f/f*^ mice were used to create gene regulatory networks using software GENIE3 (version 1.12.0). Cytoscape (version 3.8.2) was used to visualize the TF regulatory network.

### Cell–cell communication analysis

Cell–cell interaction analysis was conducted using the scRNA-seq data and the CellChat R package (version 1.1.0) (www.cellchat.org). We evaluated the average expression of each ligand-receptor pair in club cells, goblet cells, resident macrophages, and monocyte-derived macrophages, and only those with a *p* value lower than 0.05 were used for predicting the potential cell–cell interactions that were associated with Lkb1. When the ligand or the receptor was undetectable, a cell–cell interaction was considered nonexistent.

### Statistical analysis

Data are expressed as the mean ± SEM. The significance of the results was assessed using an unpaired *t* test between the two groups. Results were considered statistically significant at a *p* ≤ 0.05.

## Results

### Patients with COPD exhibit a decreased LKB1 expression in the airway epithelium

**Fig. 1:**
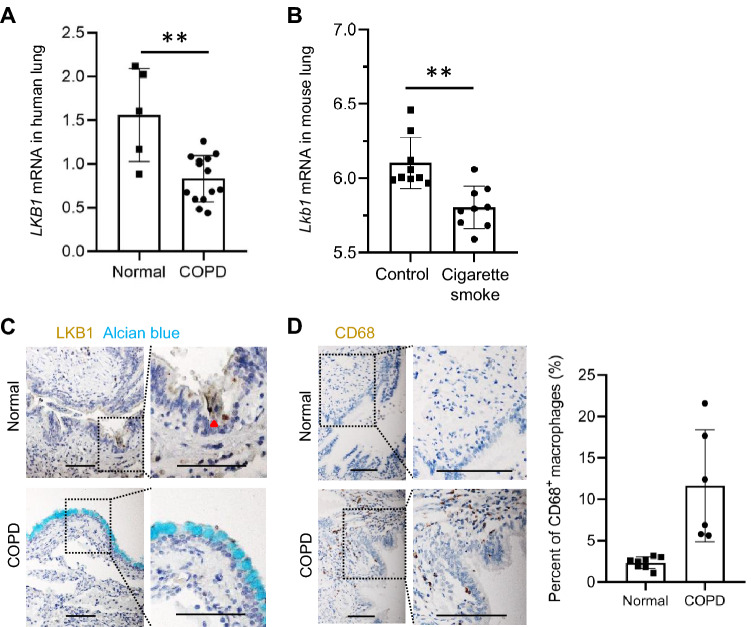
LKB1 expression is downregulated in the lungs of patients with COPD. **a** *LKB1* transcript expression in lung homogenates of normal healthy subjects or patients with COPD. **b** *LKB1* transcript expression in lung homogenates of control mice or cigarette smoke-exposed mice. **c** Immunohistochemistry staining of LKB1 and Alcian blue staining of the lung sections of normal subjects and subjects with COPD. **d** Immunohistochemistry analysis of CD68 staining of the lung sections of normal subjects and subjects with COPD. Quantification of the CD68-positive cells on the lung sections of normal subjects and subjects with COPD

We retrieved the transcriptomic data from 14 patients with COPD and 5 healthy controls from publicly available GEO datasets (GSE106986) [34]. Our analyses revealed that the expression of LKB1 transcripts was significantly reduced in the lung homogenates from patients with COPD than in those from normal controls (Fig. 1a). Reanalysis of the retrieved transcriptomic data from cigarette smoke-exposed mice also showed a decrease in Lkb1 expression in the lungs (GSE125521) (Fig. 1b). Immunohistochemistry staining also showed an evident LKB1 level reduction in the lung epithelium in patients with COPD (Fig. 1c). Consistent with previous findings [35, 36], goblet metaplasia and macrophage accumulation were observed in subjects with COPD (Fig. 1c, d). However, as compared with the controls, LKB1 expression remained unchanged in the airway epithelial cells from patients with asthma or in the lung tissues from house dust mite-exposed mice based on our reanalysis of the retrieved transcriptomic data (GSE67472; GSE71822) (Figure S1A, B). We retrieved and reanalyzed the publicly available scRNA-seq data of airway epithelial cells from patients with cystic fibrosis (GSE150674). We found that compared with normal healthy controls, the LKB1 transcript level remained unchanged in the airway epithelial cells from patients with cystic fibrosis (Figure S2).

### *Lkb1* knockout in lung epithelium promotes goblet cell metaplasia

**Fig. 2:**
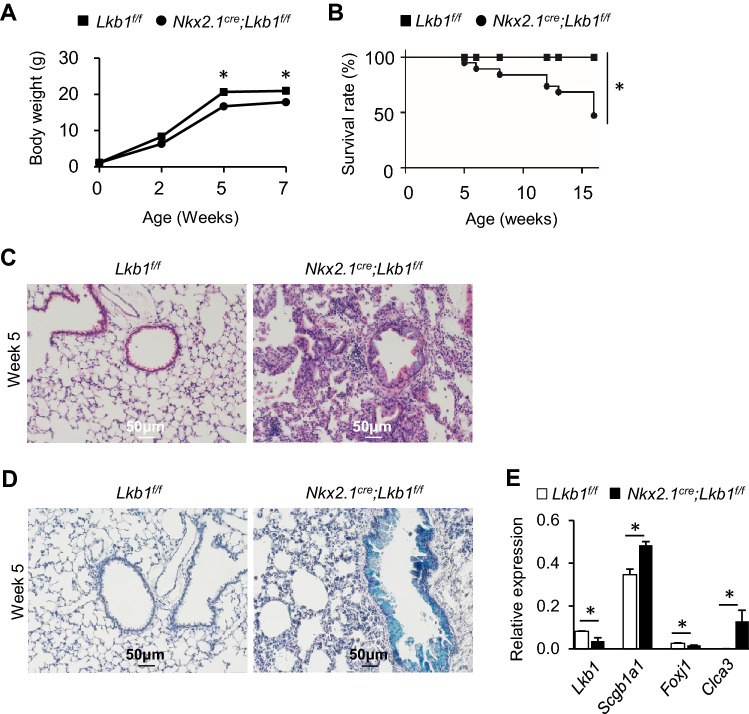
Epithelial loss of *Lkb1* leads to airway goblet cell metaplasia and inflammation. **a** The body weight of *Nkx2.1*^*Cre*^*; Lkb1*^*f/f*^ and *Lkb1*^*f/f*^ mice was measured at various time points after birth (*n* = 5–6 per group). **b** Survival analysis of *Nkx2.1*^*Cre*^*; Lkb1*^*f/f*^ and *Lkb1*^*f/f*^ mice after birth (*n* = 18–23). **c** Hematoxylin and eosin staining indicated inflammatory infiltration and epithelial alterations in *Nkx2.1*^*Cre*^*; Lkb1*^*f/f*^ mice after five postnatal weeks (*n* = 5). **d** Alcian blue staining showing airway goblet cell metaplasia in *Nkx2.1*^*Cre*^*; Lkb1*^*f/f*^ mice after five postnatal weeks (*n* = 5). **e** Quantitative PCR analysis of the indicated genes in the lungs isolated from *Nkx2.1*^*Cre*^*; Lkb1*^*f/f*^ and control mice after 5 postnatal weeks (*n* = 3). Data are representative of two or more independent experiments with the error bars representing the mean ± SD. *p < 0.05

To investigate whether goblet cell differentiation is regulated by LKB1, we crossed *Lkb1*^*f/f*^ mice with *Nkx2.1*^*Cre*^ mice to generate *Nkx2.1*^*Cre*^*; Lkb1*^*f/f*^ mice, in which LKB1 was specifically deleted in the embryonic and adult lung epithelia. Our findings revealed that the general outlook of *Nkx2.1*^*Cre*^*; Lkb1*^*f/f*^ mice was similar to that *of Lkb1*^*f/f*^ littermate controls at postnatal day 1 (Figure S3A). At this stage, there was no difference in the body and lung weight between *Nkx2.1*^*Cre*^*; Lkb1*^*f/f*^ and *Lkb1*^*f/f*^ mice (Figure S3B). However, compared with the controls, airway epithelial thickening was evident in *Nkx2.1*^*Cre*^*; Lkb1*^*f/f*^ mice (Figure S3C). This was later demonstrated to be related to an increased number of CCSP-expressing club cells (Figure S3D, E). Goblet cells were not observed in *Nkx2.1*^*Cre*^*; Lkb1*^*f/f*^ mice (data not shown). However, we noticed that by postnatal week 2, *Nkx2.1*^*Cre*^*; Lkb1*^*f/f*^ mice were smaller and had gained 10–25% less weight than the control littermates (Fig. 2a). By week 5 after birth, the *Nkx2.1*^*Cre*^*; Lkb1*^*f/f*^ mice had died (Fig. 2b). Histological examination of the lungs showed massive inflammatory cell infiltration in the peribronchiolar connective tissues, in addition to the thickening of the airway epithelium (Fig. 2c). Compared with the control mice, Alcian blue staining revealed a marked increase in the number of mucous cells within the bronchi of *Nkx2.1*^*Cre*^*; Lkb1*^*f/f*^ mice (Fig. 2d). *Foxj1* expression was decreased in the absence of LKB1, as revealed by the RT-qPCR analysis of whole lungs (Fig. 2e). However, compared with the controls, the expression of *Scgb1a1* and *ClCa3* was significantly increased in the lungs of *Nkx2.1*^*Cre*^*; Lkb1*^*f/f*^ mice (Fig. 2e). These data suggest that Lkb1 restricts goblet cell differentiation in the lungs.

**Fig. 3:**
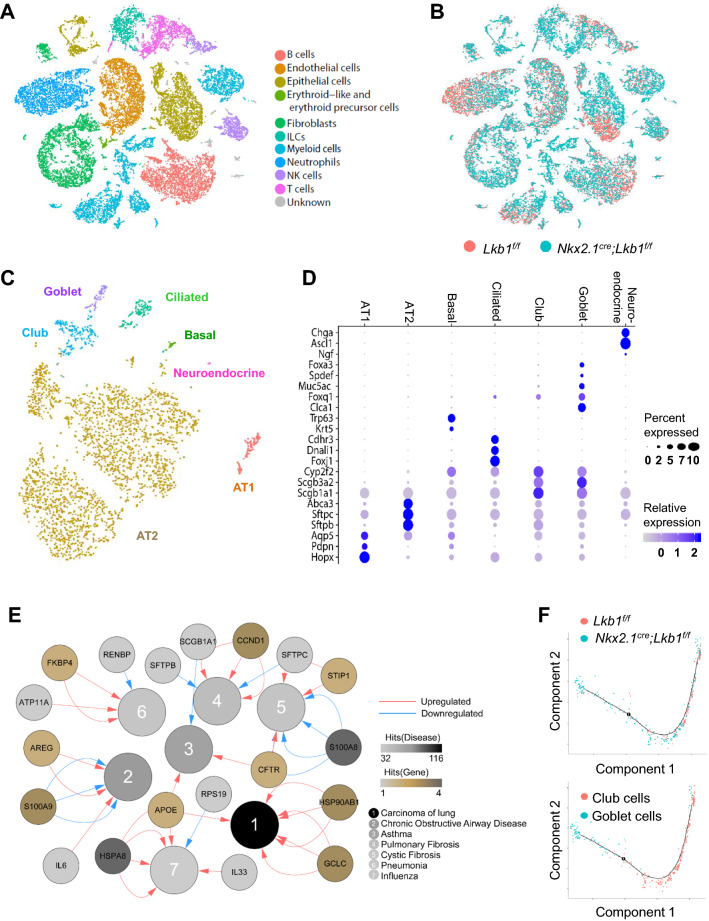
Single-cell RNA-seq analysis demonstrating the stimulated goblet cell differentiation. **a**, **b** tSNE plot of 30,408 lung cells pooled from each group (3 mice per group), color-coded by their associated cell lineages **a** or the origin of the sample type **b**. **c** tSNE plot of 3,373 lung epithelial cells, color-coded by their associated cell types. **d** Enrichment of the gene expression in each lung epithelial type. Node size is proportional to the percentage of cells in the cluster expressing a gene. Node color is proportional to the average expression level for the gene in the cluster. **e** Network plot revealing the expression of genes that changed between the epithelial cells of *Nkx2.1*^*Cre*^*; Lkb1*^*f/f*^ mice and those of *Lkb1*^*f/f*^ mice and the genes in the lung disease database (https://www.disgenet.org/home/). The red connecting lines show the upregulated genes and the blue connecting lines show the downregulated genes in the epithelial cells of *Nkx2.1*^*Cre*^*; Lkb1*^*f/f*^ mice compared with those of *Lkb1*^*f/f*^ mice. The number of connecting lines represents the number of epithelial cell types that express these genes. The color of the gene nodes (from white to dark brown) represents the number of epithelial cell types (from low to high), respectively. The lung disease types are listed in order. The color of the disease number (from white to black) represents the number of genes related to the indicated disease (from low to high), respectively. **f** Predicted effect of *Lkb1* on the differentiation model of club cells into goblet cells, and predicted differentiation lineage model between club and goblet cells

To characterize the mechanisms underlying LKB1-deficiency-induced lung pathology, we performed single-cell RNA-seq analysis of the lungs of *Nkx2.1*^*Cre*^*; Lkb1*^*f/f*^ and *Lkb1*^*f/f*^ mice. In total, 30,408 lung cells were collected for the scRNA-seq analysis of the whole lung cells isolated from 5-week-old *Nkx2.1*^*Cre*^*; Lkb1*^*f/f*^ or *Lkb1*^*f/f*^ mice, when the *Nkx2.1*^*Cre*^*; Lkb1*^*f/f*^ mice began to die (Fig. 3a, b). We used established markers to validate the cell types in each cluster (Figs. 3a and S4). We employed 2D graphs to reveal the diverse cell lineages, including the epithelium (expressing *epithelial cellular adhesion molecule* (*Epcam*)), endothelium (*cadherin 5* (*Cdh5*), *Myc target protein 1* (*Myct1*)), fibroblasts (*collagen type I alpha 2 chain* (*Col1a2*), *osteoglycin* (*Ogn*)), myeloid cells (triggering receptor expressed on myeloid cells 2 (*Trem2*)), neutrophils (resistin-like gamma precursor (*Retnlg*), matrix metallopeptidase 9 (*Mmp9*)), erythroid-like and erythroid precursor cells (5′-aminolevulinate synthase 2 (*Alas2*)), lymphoid lineages which separated into B cells (*CD19*), ILC (interleukin 1 receptor-like 1 (*Il1rl1*)), natural killer cells (C–C motif chemokine ligand 5 (*Ccl5*)*,* killer cell lectin-like receptor, family E, member 1 (*Klre1*)), and T cells (*CD3d*) (Figure S4). Overall, based on the single-cell map that assigned cell types, the cellular differentiation and transcriptomic profiles of the lungs were further studied.

We further used marker genes to enhance the annotation of the epithelial clusters, generating seven epithelial subpopulations, including AT2 cells (expressing known marker ATP-binding cassette subfamily A member 3 (*Abca3*)*,* surfactant protein C (*Sftpc*)), AT1 cells (aquaporin 5 (*Aqp5*), podoplanin (*Pdpn*)), basal cells (tumor protein 63 (*Trp63*), keratin 5 (*Krt5*)), club cells (cytochrome P450 2F2 (*Cyp2f2*), *Scgb1a1*, ciliated cells (cadherin-related family member 3 (*Cdhr3*), forkhead box J1 (*Foxj1*)), goblet cells (*Foxa3, Spdef*), and neuroendocrine cells (chromogranin A (*Chga*), *achaete-scute homolog 1* gene (*Ascl1*)) (Fig. 3c, d).

A comparison of the network plot of the DEGs of lung epithelial cells between *Nkx2.1*^*Cre*^*; Lkb1*^*f/f*^ and littermate controls showed that the epithelial-derived LKB1-relevant lung diseases were ranked by lung carcinoma, as expected, since LKB1 is known as a tumor suppressor (Fig. 3e). Among the 30 *Nkx2.1*^*Cre*^*; Lkb1*^*f/f*^ mice investigated, we found one in which lung carcinoma was developing (Figure S5). Other relevant diseases included COPD, pulmonary fibrosis, pneumonia, and influenza (Fig. 3e). Pseudotime analysis revealed the differentiation trajectories of club cells to goblet cells and showed that almost all of the goblet cells mapped to the club cell differentiation trajectory were derived from *Nkx2.1*^*Cre*^*; Lkb1*^*f/f*^ mice, confirming the inhibitory role of LKB1 in goblet cell differentiation (Fig. 3f).

**Fig. 4:**
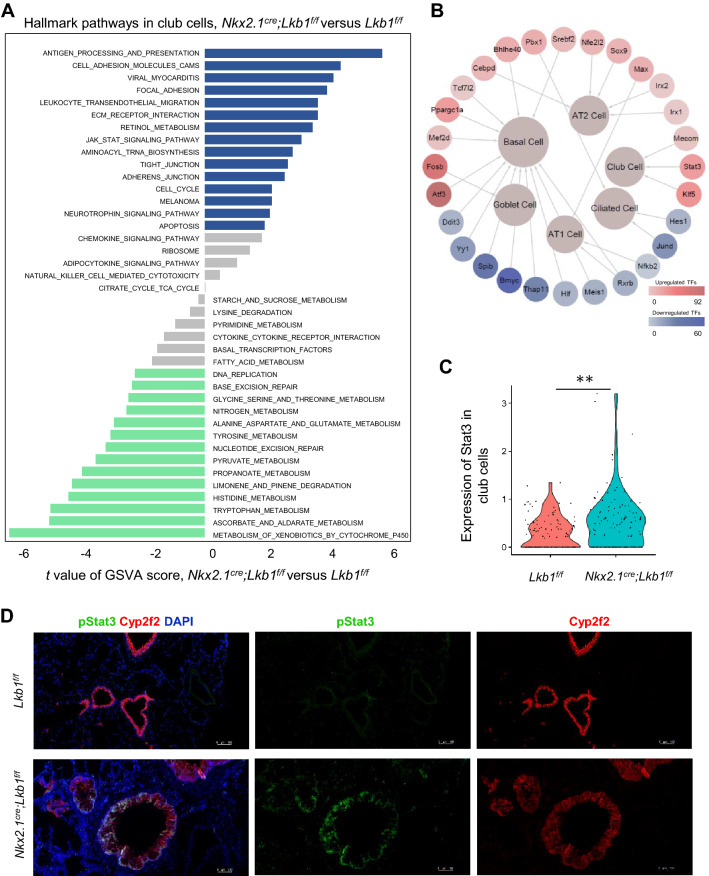
Molecular effects of *Lkb1* knockdown in club cells. **a** Discrepancies in pathway activities scored using GSVA, between the club cells from *Nkx2.1*^*Cre*^*; Lkb1*^*f/f*^ mice versus those from *Lkb1*^*f/f*^ mice. **b** Transcriptional network revealing the transcription factors (TFs) identified in the epithelium of *Nkx2.1*^*Cre*^*; Lkb1*^*f/f*^ mice versus *Lkb1*^*f/f*^ mice via SCENIC. The outer nodes represent the upregulated and downregulated TFs (in red and blue, respectively). The color of the TFs (from light to dark) reveals the number of target genes regulated by the TFs (from low to high), respectively. The inner nodes indicate the subpopulations of lung epithelium and the sizes of the nodes represent the number of target genes involved in this subpopulation. **c** Violin plots of Stat3 expression in the club cells of *Nkx2.1*^*Cre*^*; Lkb1*^*f/f*^ mice compared with those of *Lkb1*^*f/f*^ mice. **d** Immunofluorescent staining of the lung sections from *Nkx2.1*^*Cre*^*; Lkb1*^*f/f*^ mice versus those from *Lkb1*^*f/f*^ mice with phosphorylated STAT3 and Cyp2f2 (*n* = 3)

KEGG pathway signatures and SCENIC analysis suggested that the Janus kinase/signal transducers and activators of transcription (JAK/STAT) signaling pathway may be affected by *Lkb1* (Fig. 4a, b). The *Stat3* mRNA levels were elevated in LKB1-deficient club cells compared to those in normal club cells (Fig. 4b, c). Consistently, immunofluorescence staining verified the upregulation of phosphorylated STAT3 (pSTAT3) in club cells in the absence of LKB1 (Fig. 4d). These data suggest that STAT3 may be involved in club cell fate determination.

### *Lkb1* silencing upregulates RELM-α in club cells that regulate goblet cell differentiation

**Fig. 5:**
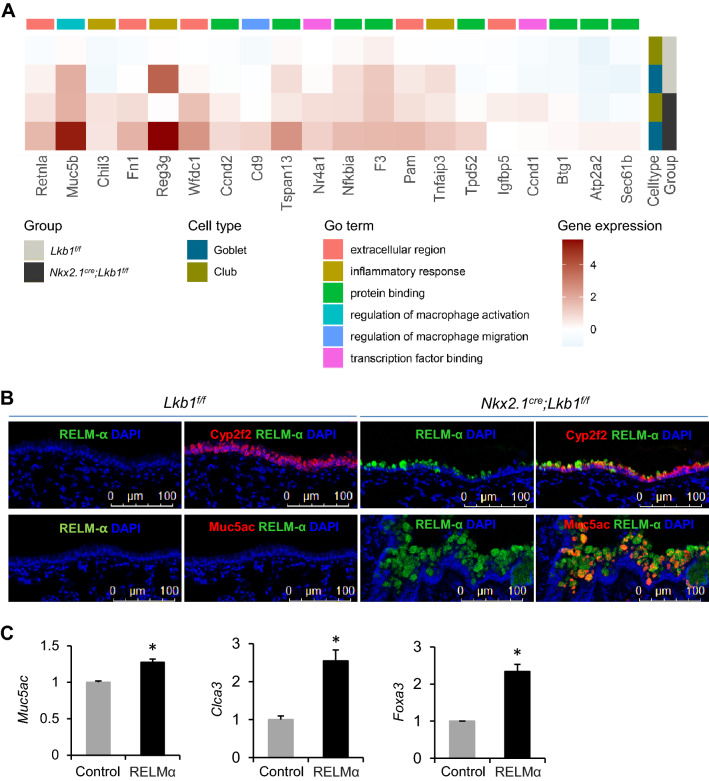
*Lkb1* knockdown promotes RELM-α, which drives club cell differentiation into goblet cells. **a** Heatmap revealing the co-upregulated DEGs of club cells and goblet cells which are related to the extracellular region, inflammation, and regulation of macrophages in *Nkx2.1*^*Cre*^*; Lkb1*^*f/f*^ mice versus *Lkb1*^*f/f*^ mice. **b** Immunofluorescent staining of the lung sections from *Nkx2.1*^*Cre*^*; Lkb1*^*f/f*^ mice versus *Lkb1*^*f/f*^ mice with Cyp2f2 and RELM-α or with Muc5Ac and RELM-α (*n* = 3). **c** Gene expression analysis of the indicated transcripts using RT-qPCR (relative to β-actin) in the organoid cultures of club cells in the presence or absence of RELM-α (*n* = 5). Data are representative of two or more independent experiments with the error bars representing the mean ± SD. *p < 0.05

To investigate the molecular mechanism of LKB1 in the regulation of club cell differentiation into goblet cells, we compared the transcriptomic profiles of club cells in the scRNA-seq data from *Nkx2.1*^*Cre*^*; Lkb1*^*f/f*^ mice and in the littermate controls using scRNA-sequencing. We observed 136 differentially expressed transcripts with fold changes of over 1.5-fold with 113 upregulated and 23 downregulated transcripts. The top upregulated transcripts in the club cells of *Nkx2.1*^*Cre*^*; Lkb1*^*f/f*^ mice included resistin-like molecule alpha (*Retnla*) and chitinase-like protein 3 (*Chil3*) (Figure S6A, Table S1). The increase in *Retnla* and *Chil3* expression was confirmed via bulk RNA-seq analysis of the lung epithelial cells sorted from *Nkx2.1*^*Cre*^*; Lkb1*^*f/f*^ mice and the littermate controls (Figure S6B, Table S2). The transcriptomic profiles identified 120 upregulated and 77 downregulated transcripts in goblet cells in the absence of LKB1 (Figure S6C). The elevated transcripts included Clca1 (79-fold), Agr2 (fivefold), and Muc5b (fivefold), which are related to mucus production or secretion (Table S3). We found that the *Retnla* expression levels were increased in both club cells and goblet cells in the absence of LKB1, along with the upregulation of mucus-related and inflammation-related genes, such as *Muc5b*, *Chil3*, *Cd9*, and *Tnfaip3* (fold change > 1.5; *p* < 0.05) (Fig. 5a). These data suggest that *Retnla* may regulate goblet cell differentiation.

Confocal microscopy revealed that RELM-α, the protein encoded by *Retnla*, was undetectable in normal mouse club and goblet cells (Fig. 5b). However, RELM-α was found to be upregulated and co-localized with Cyp2F2 (club cell marker) and Muc5Ac (goblet cell marker) in the lungs of *Nkx2.1*^*Cre*^*; Lkb1*^*f/f*^ mice (Fig. 5b). Airway goblet cells expressed lower levels of Cyp2F2 than club cells, in *Nkx2.1*^*Cre*^*; Lkb1*^*f/f*^ mice (Figure S7). Immunostaining of the adjacent lung sections suggested the presence of two types of goblet cells: one expressing RELM-α and the other expressing little or no RELM-α (Figure S7). These data suggest that LKB1 inhibits the expression of RELM-α in club cells.

To examine whether RELM-α modulates goblet cell differentiation in general, we implemented an in vitro 3D organoid culture established previously [6]. Club cells characterized by a CD31^−^CD34^−^CD45^−^EpCAM^+^ Sca1^+^CD24^low^ phenotype were isolated from C57BL/6 mice via FACS, followed by an organoid culture with supportive lung fibroblast cells (Figure S8A, B). The size and forming efficiency of club cell-derived organoids remained unchanged in the presence of recombinant RELM-α (Figure S8B–D), suggesting that RELM-α plays little role in club cell proliferation. However, the expression of the goblet cell markers Muc5Ac, ClCa3, and Foxa3 was promoted in organoid cultures supplemented with recombinant RELM-α based on qRT-PCR analysis (Fig. 5c). To confirm the role of Lkb1 in goblet cell differentiation, we established *Scgb1a1*^*CreER*^*; Lkb1*^*f/f*^ mice which exhibited conditional loss of Lkb1 in airway club cells after tamoxifen injection (Figure S9A). As predicted, the organoid cultures of mouse airway club cells indicated that *Clca3* gene expression was promoted in absence of Lkb1 (Figure S9B). The results of the protein–protein interaction prediction tool STRING or GeneMANIA analysis showed no evidence of the interaction between Lkb1 and RELM-α (Figure S10, 11). Taken together, these data suggest that RELM-α promotes goblet cell differentiation.

### Epithelial silencing of *Lkb1* induces macrophage accumulation and activation

**Fig. 6:**
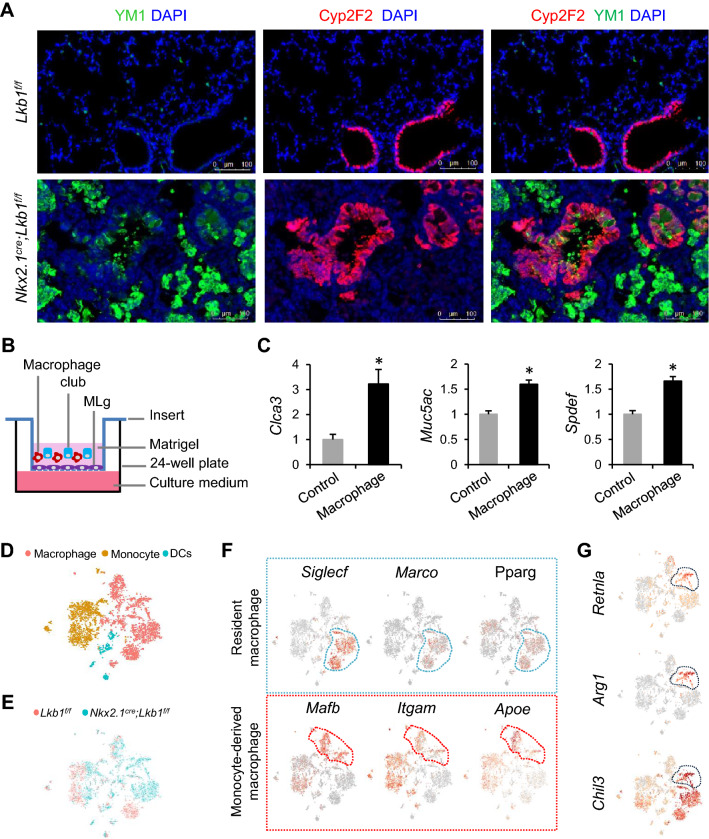
Segregated macrophages around airway club cells in *Nkx2.1*^*Cre*^*; Lkb1*^*f/f*^ mice. **a** Immunofluorescent staining of the lung sections from *Nkx2.1*^*Cre*^*; Lkb1*^*f/f*^ mice versus *Lkb1*^*f/f*^ mice with Cyp2f2 and YM1 (*n* = 3). **b** Schematic illustration of the macrophage-club co-culture organoid model. **c** Quantitative PCR analysis of the indicated transcripts (relative to β-actin) in the organoid cultures of mouse club cells with or without macrophages (*n* = 5). **d**, **e** tSNE plot of 5051 lung myeloid cells, color-coded by their associated cell lineages **d** or the origin of the sample type **e**. **f** Existing markers identifying the source of the resident or monocyte-derived macrophages in the lungs. **g** Known markers showing that part of the monocyte-derived macrophages are of an alternatively activated phenotype. Data are representative of two or more independent experiments with the error bars representing the mean ± SD. *p < 0.05

RELM-α drives the recruitment of monocytes and promotes the alternative activation of pulmonary macrophages [21, 37–39]. To examine whether RELM-α upregulation results in the accumulation and activation of macrophages, we performed immunofluorescent staining of lung sections using antibodies against chitinase-like 3 (Chil3/Ym1), Muc5Ac, and Cyp2F2. We observed that the Ym1-positive macrophages aggregated around the airway Cyp2F2-positive cells (Fig. 6a). Confocal immunofluorescent staining results demonstrated that the majority of the Muc5Ac-positive goblet cells were stained with Ym1, whereas no immunoreactive Ym1 was found in the Cyp2f2-hi club cells of *Nkx2.1*^*Cre*^*; Lkb1*^*f/f*^ mice (Figure S12A, B). To examine whether macrophages affect the differentiation of club cells into goblet cells, we isolated CD11b^+^ macrophages from lung tissues of C57BL/6 mice by FACS and co-cultured them with club cells at a 1:2 ratio (Figs. 6b, S13). Quantitative PCR analysis indicated that the expression of goblet cell markers *Clca3*, *Muc5ac*, *Spdef* was increased in organoid cultures in presence of macrophages (Fig. 6c).

Next, we characterized these macrophages based on single-cell RNA-seq data analysis with marker genes (Figure S14). Lung myeloid cells can be segregated into three distinct cell types: dendritic cells (interferon regulatory factor 8 [*Irf8*], C-type lectin domain containing 9A [*Clec9a*]), monocytes (adhesion G protein-coupled receptor E4 (*Adgre4*)), or macrophages (mannose receptor C-type 1 (*Mrc1*), heme oxygenase 1 (*Hmox1*)) (Figs. 6d, S14). Consistent with the findings mentioned above (Fig. 6a), the macrophage population was more abundant in the lungs of *Nkx2.1*^*Cre*^*; Lkb1*^*f/f*^ mice than in those of control mice (Fig. 6e). To explore the origin of these macrophages, we used established cell markers that have been reported as tissue-resident or monocyte-derived macrophage markers to segregate the macrophage clusters [40–42]. Lung resident macrophages were identified as clusters expressing higher levels of sialic acid-binding Ig-like lectin F (*Siglecf*), macrophage receptor with collagenous structure (*Marco*), and peroxisome proliferator-activated receptor-gamma (*Pparg*), and monocyte-derived macrophages were identified as clusters expressing high levels of MAF BZIP TF B (*Mafb*), integrin subunit alpha M (*Itgam*), and apolipoprotein E (*Apoe*) (Fig. 6f). Some monocyte-derived macrophages expressed *Retnla*, *Arg1*, and *Chil3*, which is indicative of their alternatively activated M2 phenotype (Fig. 6g). M2 macrophages were observed in *Nkx2.1*^*Cre*^*; Lkb1*^*f/f*^ mice, but not in control mice (Fig. 6e). Collectively, these data suggest that the suppression of epithelial LKB1 induces the recruitment of monocytes to the lungs and promotes their M2 phenotype activation.

**Fig. 7:**
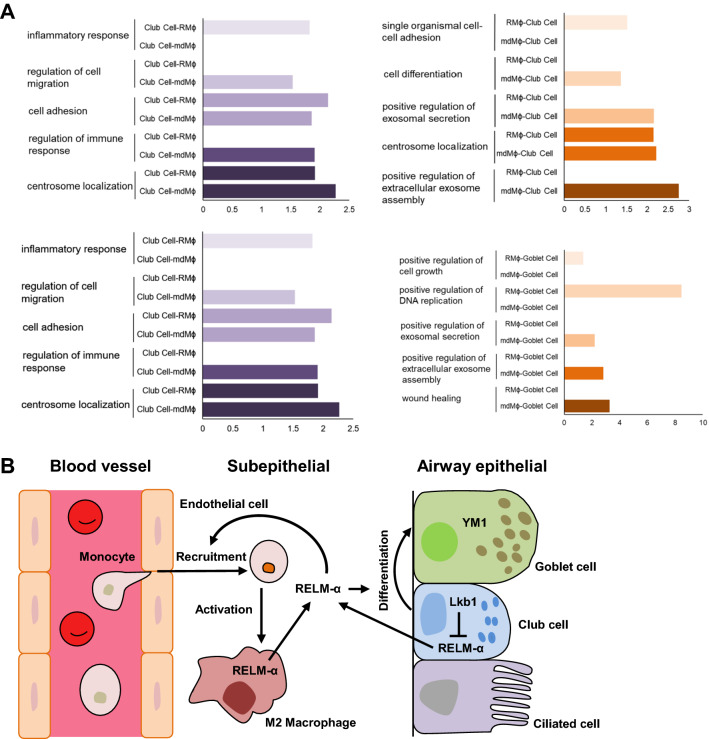
Lkb1 mediated cell–cell interactions among club cells, goblet cells, and macrophages. **a** Gene ontology enrichment analyses based on the differences in the receptor and ligand networks among club cells, goblet cells, and macrophages. **b** Schematic expression of the LKB1-mediated crosstalk between epithelial progenitor cells and macrophages in goblet cell differentiation

To further understand how the origin of macrophages affects club cell differentiation in the absence of Lkb1, we analyzed the cell–cell interactions between macrophages and club cells, as well as between macrophages and goblet cells, and found that club cells interact with monocyte-derived macrophages in different ways from those with the resident macrophages (Fig. 7a). For instance, monocyte-derived macrophages have a greater impact on the differentiation of mouse club cells (Fig. 7a). Altogether, these data suggest that LKB1 is an inhibitory factor in regulating airway goblet cell differentiation. *Lkb1* loss may upregulate the airway progenitor cell expression of RELM-α, which promotes airway goblet cell differentiation and pulmonary macrophage infiltration (Fig. 7b). By secreting RELM-α, monocyte-derived macrophages differentiate into the M2 phenotype to further enhance goblet cell fate.

## Discussion

In the present study, we observed that LKB1 restricts goblet cell differentiation in mouse airways. Under normal conditions, the presence of goblet cells in mouse airways is not evident. However, when LKB1 expression is inhibited from the embryonic stage, goblet cell metaplasia develops in the adult stage. In the absence of LKB1, the level of monocyte attracting factor RELM-α is increased in club cells and promotes goblet cell differentiation. Macrophages are also found to be in close proximity to goblet cell metaplasia. ScRNA-seq analysis also indicated the monocytic origin of most infiltrated macrophages. Our data indicate that monocyte-derived macrophages interact with club cells and contribute to goblet cell differentiation and monocyte recruitment to the lungs. We observed a downregulation of LKB1 and macrophage accumulation in subjects with COPD, suggesting a possible role of LKB1 in goblet cell metaplasia in COPD pathogenesis.

LKB1 is widely expressed in human adult tissues and serves as a suppressor of neoplastic transformation. Deletion, mutation, or a reduced expression of LKB1 has been demonstrated to increase the risk of malignant tumors in lungs, colon, breast, cervical, gastrointestinal, and pancreatic tissues through AMP-activated protein kinase (AMPK)-mediated metabolic reprogramming [43]. LKB1 maintains stem cell quiescence and survival, restricting the entry of stem cells into the cell cycle [44, 45]. Airway branching and normal ciliated cell differentiation require LKB1 [32, 46]. However, Lkb1 represses the differentiation of intestinal stem cells into secretory lineages and mucin production by secretory cells [47, 48]. Similar to the intestine, we observed that the silencing of epithelial Lkb1 causes goblet cell metaplasia and mucus overproduction through RELM-α in the lungs. RELM-α plays a role in the recruitment of monocytes/macrophages [21, 37, 49]. An uncontrolled goblet cell differentiation is a prominent feature of a number of respiratory diseases, including COPD and asthma. Mucin hypersecretion prevents mucociliary clearance and reduces gas exchange. We found that the *LKB1* transcript levels were lower in the airway epithelium of patients with COPD than in healthy subjects. None of the existing medicines for COPD, including bronchodilators and anti-inflammatory steroids, targets goblet cell differentiation and mucus secretion [50]. Therefore, these medicines suppress COPD symptoms without treating the condition. Therefore, restoring LKB1 expression in airway progenitor cells is a potential therapeutic strategy to tackle COPD by limiting goblet cell differentiation and mucus hypersecretion. It is important to investigate whether the existing drugs for COPD impair LKB1 expression in airway epithelial progenitor cells.

In addition to the direct role of LKB1 in limiting goblet cell differentiation, it indirectly controls goblet cell differentiation by affecting the crosstalk between club cells and macrophages. Club-macrophage interactions have been suggestive but the underlying mechanisms remain largely unknown. The lack of CCSP has been associated with COPD development [51], and recombinant rat CCSP treatment was shown to lead to a macrophage number decrease [52]. The recruitment and activation of macrophages are normally observed in the lungs of patients with COPD [53]. A previous report demonstrated that the inhibition of Lkb1 promotes the recruitment of monocytes and macrophages in the kidneys [54]. We observed that monocyte-derived macrophages were adjacent to airway progenitor cells in the absence of LKB1 in the lungs. These macrophages are monocyte-derived and display an alternative M2 phenotype with RELM-α expression, that favors the differentiation of club cells into goblet cells. Moreover, M2 macrophages are also prominent in the lungs of patients with COPD [55]. Our data suggest that M2 macrophages direct airway epithelial progenitor cell fate by detouring to the goblet cell phenotype. The elimination of lung M2 macrophages may improve goblet cell metaplasia in COPD. However, the resident macrophages play different roles in airway epithelial stem/progenitor cell fate specification since they interact with club cells in different ways from those with monocyte-derived macrophages. Dagher et al. showed that IL33 in resident M2 macrophages favors club cell regeneration [56]. Additionally, it is also known to induce goblet cell differentiation [57, 58].

RELM-α has been reported to be upregulated in the epithelial cells of asthmatic mouse lungs [59], in which goblet cell metaplasia occurs persistently. The increase in the RELM-α level in airway epithelial cells in the Alternaria-induced asthmatic mouse model was STAT6-dependent [21], while that in the oncostatin M-induced mouse model did not require STAT6 [39]. These studies suggest that RELM-α affects airway goblet cell differentiation through multiple pathways. RELM-α is regulated by STAT6 in macrophages [60]. However, it remains unknown whether STAT3 modulates RELM-α expression. STAT3 was previously shown to be associated with the expression of mucus-associated molecules in the intestine [61]. In this study, the hallmark pathway gene signature and SCENIC predicted STAT3 to be a TF responsible for goblet cell hyperplasia in the lungs. We detected an increased phosphorylated STAT3 level, in line with recent studies, confirming that an increased airway goblet cell hyperplasia may be accompanied by an elevated activation of STAT3 [62, 63].

This study has several limitations. First, airway goblet hyperplasia is also a feature of asthma, but our analysis of the retrieved transcriptomic data indicated that LKB1 expression was not altered in the lungs of asthma patients or cigarette smoke-exposed mice. It remains unknown whether a LKB1 downregulation is associated with asthma/COPD overlap pathology. Second, *Nkx2.1*^*Cre*^*; LKB1*^*f/f*^ mice started to develop airway goblet cell hyperplasia at 2 weeks and died at the age of 5 weeks (equivalent to 30 years of human age), while COPD commonly occurs in people over 40 years old. It is not known how age impacts Lkb1 expression in healthy subjects or in patients with COPD. Third, the activation of AMP-activated kinase, a downstream target of LKB1, was shown to reduce excessive airway inflammation that may be beneficial to patients with cystic fibrosis. It would be worthwhile to explore whether LKB1 is developmentally defective in this genetic disease in the future since we were unable to. Fourth, our protein–protein interaction prediction results suggest that Lkb1 does not interact with RELM-α directly. Further functional experiments must be done to verify this. The sources of RELM-α were from both LKB1-deficient airway epithelial cells and M2 macrophages surrounding the epithelial cells, but the underlying mechanism requires further investigation. Future investigations on the immunological regulation of the lung epithelial stem/progenitor cell fate, especially the crosstalk between them and macrophages or other immune cells, will provide insights into the pathogenesis of COPD and the development of new therapeutic targets.

## Supplementary Information

Below is the link to the electronic supplementary material.

Supplementary file1 (DOCX 44 kb)

Supplementary file2 (DOCX 6075 kb)

## Data Availability

All data and material used in the study are available.

## Abbreviations

LKB1: Liver kinase B1
COPD: Chronic obstructive pulmonary disease
AT2: Alveolar epithelial type II cells
AT1: Alveolar epithelial type I cells
ILC: Innate lymphoid cell
DC: Dendritic cell
NK: Natural killer
ScRNA-seq: Single-cell RNA sequencing
