# Supplementary material for: LKB1 deficiency upregulates RELM-α to drive airway goblet cell metaplasia
Source: Cell Mol Life Sci. 2021 Dec 18;79(1):42. doi: 10.1007/s00018-021-04044-w (PMC8738459; doi:10.1007/s00018-021-04044-w)

**LKB1 deficiency upregulates RELM-α to drive airway goblet cell metaplasia**

Yu Li^1,2,3,4,#^, Qiuyang Zhang^1,2,3,4,#^, Li Li^5,#^, De Hao^1^, Peiyong Cheng^1^, Kuan Li^1,2,3,4^, Xue Li^1,2,3,4^, Jianhai Wang^1,2,3,4^, Qi Wang^2^, Zhongchao Du^2^, Hongbin Ji^6^, Huaiyong Chen^1,2,3,4,^*

^1^ Department of Basic Medicine, Haihe Hospital, Tianjin University, Tianjin, China

^2^ Key Research Laboratory for Infectious Disease Prevention for State Administration of Traditional Chinese Medicine, Tianjin Institute of Respiratory Diseases, Tianjin, China

^3^ Department of Basic Medicine, Haihe Clinical School, Tianjin Medical University, Tianjin, China

^4^ Tianjin Key Laboratory of Lung Regenerative Medicine, Tianjin, China

^5^ Department of Respiratory Medicine, Haihe Clinical School, Tianjin Medical University, Tianjin, China

^6^ State Key Laboratory of Cell Biology, CAS Center for Excellence in Molecular Cell Science, Shanghai Institute of Biochemistry and Cell Biology, Chinese Academy of Sciences, University of Chinese Academy of Sciences, Shanghai, China.

^#^ Contributed equally.

***Cellular and Molecular Life Sciences***

**Correspondence to:**

Dr. Huaiyong Chen, Department of Basic Medicine, Haihe Hospital, Tianjin University, Tianjin 300350, China, E-Mail: [huaiyong.chen@foxmail.com](mailto:huaiyong.chen@foxmail.com)

**Supplemental materials**

Supplemental figure legends

**Figure S1. Bulk RNA-seq analysis of lung tissue** (A) Analysis of *LKB1* transcript expression in lungs of normal healthy subjects or asthma patients. (E) *Lkb1* transcript expression in lungs of control mice or house dust mite (HDM)-exposed mice.


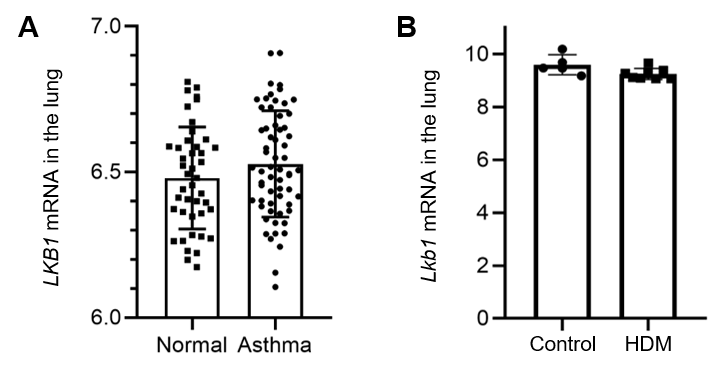


**Figure S2. Single Cell RNA Sequencing in lungs of normal healthy subjects or cystic fibrosis patients.** (A) The expression of *LKB1* transcript in total airway epithelial cells from normal subjects or patients with cystic fibrosis. (B) The expression of *LKB1* transcript in lung basal cells from normal subjects or patients with cystic fibrosis. (C) The expression of *LKB1* transcript in lung ciliated cells from normal subjects or patients with cystic fibrosis. (D) The expression of LKB1 in lung secretory cells from normal subjects or patients with cystic fibrosis.


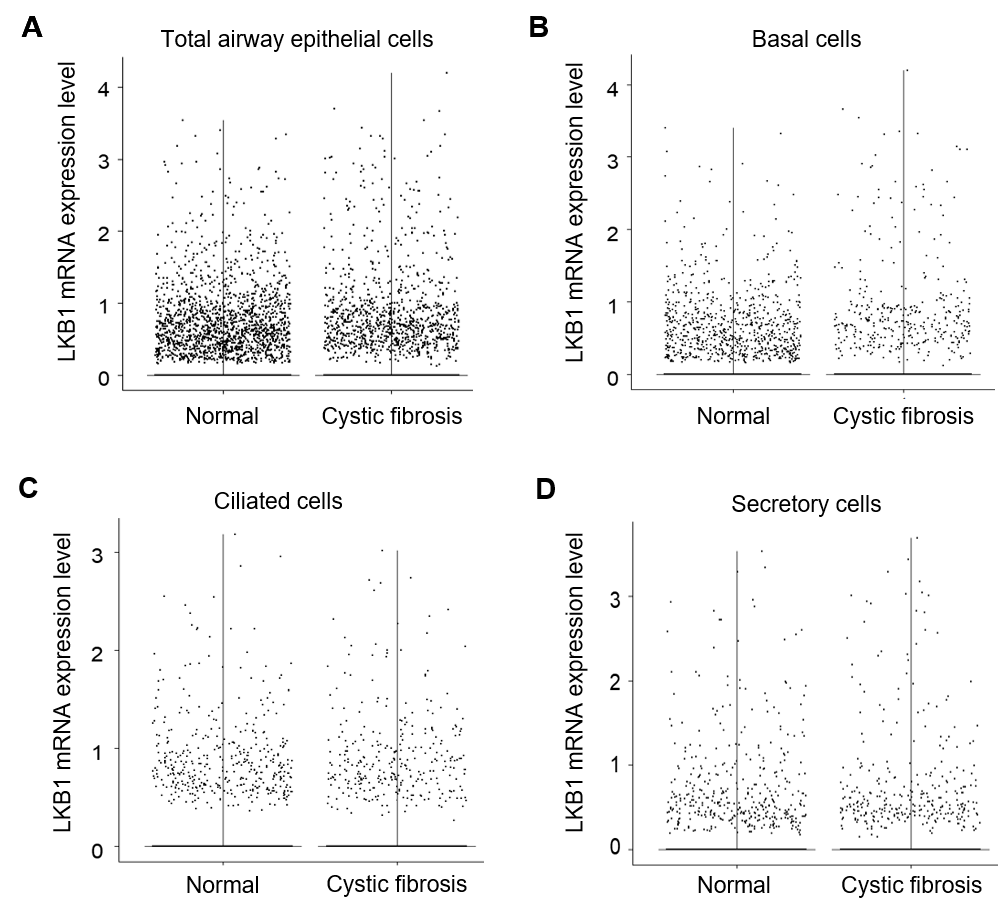


**Figure S3. Phenotype of *Nkx2.1^Cre^;Lkb1^f/f^* mice.** (A-B) *Lkb1^f/f^* and *Nkx2.1^Cre^;Lkb1^f/f^* mice were photographed and weighed at postnatal day 1 (PND1). (C) H&E staining indicated airway epithelial thickening. (D) Immunofluorescent staining of lung sections with CCSP. (E) Quantification of CCSP-positive cells in D. **p*<0.05.

**
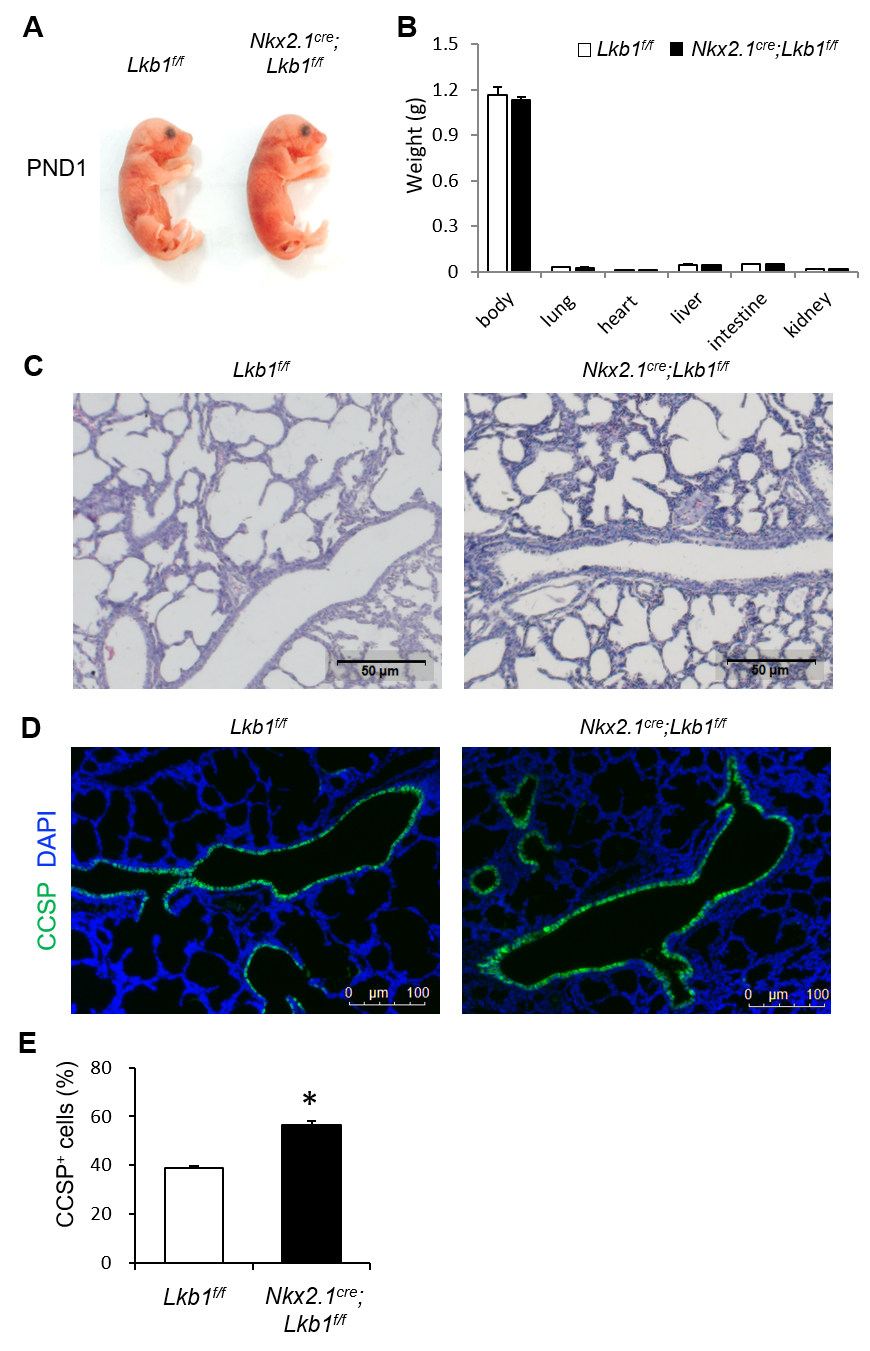
**

**Figure S4. Integrated single-cell RNA-Seq analysis of total lung cells from *Nkx2.1^Cre^;Lkb1^f/f^* and *Lkb1^f/f^* mice.** The cell markers were used to identify cell types, including epithelial cells, endothelial cells, fibroblasts, erythroid-like and erythroid precursors, B cells, T cells, ILCs, neutrophils, NK cells, and myeloid cells.


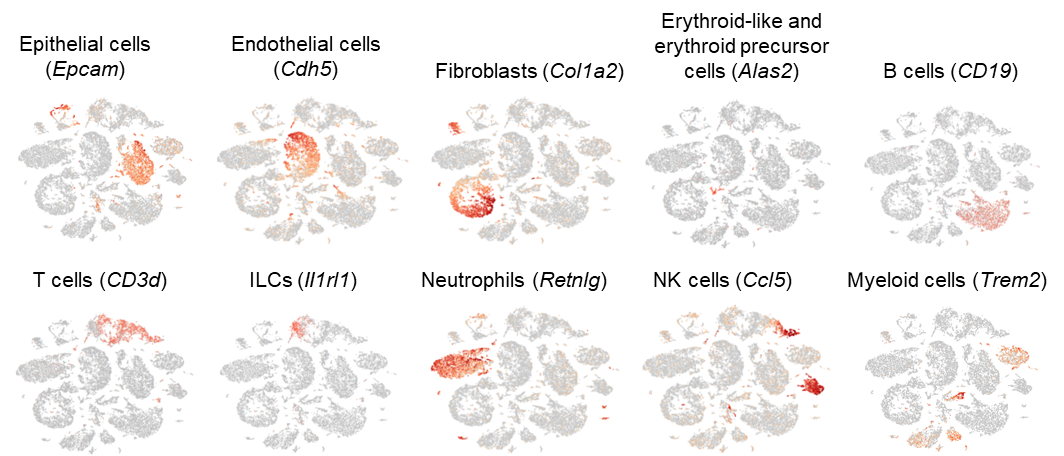


**Figure S5. The tumor formation was infrequently found in *Nkx2.1^Cre^;Lkb1^f/f^ mice*.** (A) The lung tumor appeared at the age of 21 weeks in one *Nkx2.1^Cre^;Lkb1^f/f^* mouse*.* (B) Hematoxylin and eosin staining of the lung tumor from *Nkx2.1^Cre^;Lkb1^f/f^ mice.* Scale bar, 500 μm.

**
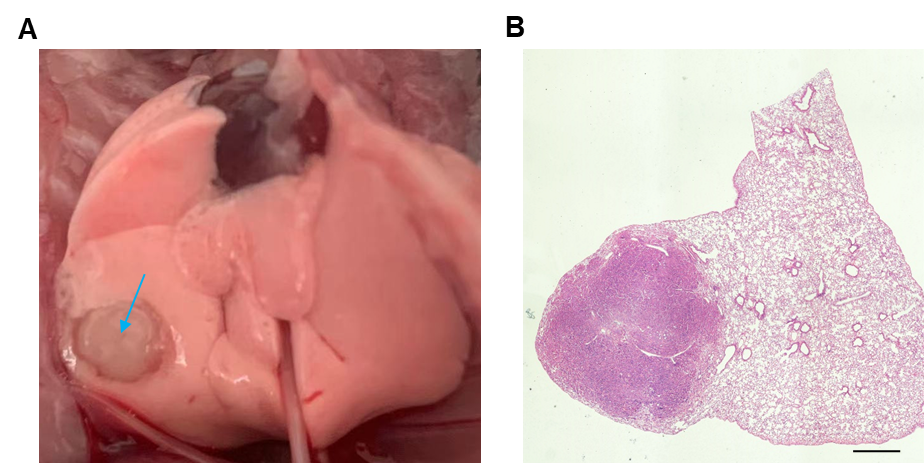
**

**Figure S6**. **Heatmap of differentially expressed transcripts in lung homogenates from *Nkx2.1^Cre^;Lkb1^f/f^* mice versus *Lkb1^f/f^* mice.** (A) Heatmap of top 25 up-regulated and 25 down-regulated transcripts in club cells from *Nkx2.1 ^Cre^;Lkb1^f/f^* mice versus *Lkb1^f/f^* mice. (B) Bulk RNA-Seq of flow cytometry-sorted epithelial cells from *Nkx2.1^Cre^;Lkb1^f/f^* mice versus *Lkb1^f/f^* mice. (C) Heatmap of top 25 up-regulated and 25 down-regulated transcripts in goblet cells from *Nkx2.1 ^Cre^;Lkb1^f/f^* mice versus *Lkb1^f/f^* mice.

**
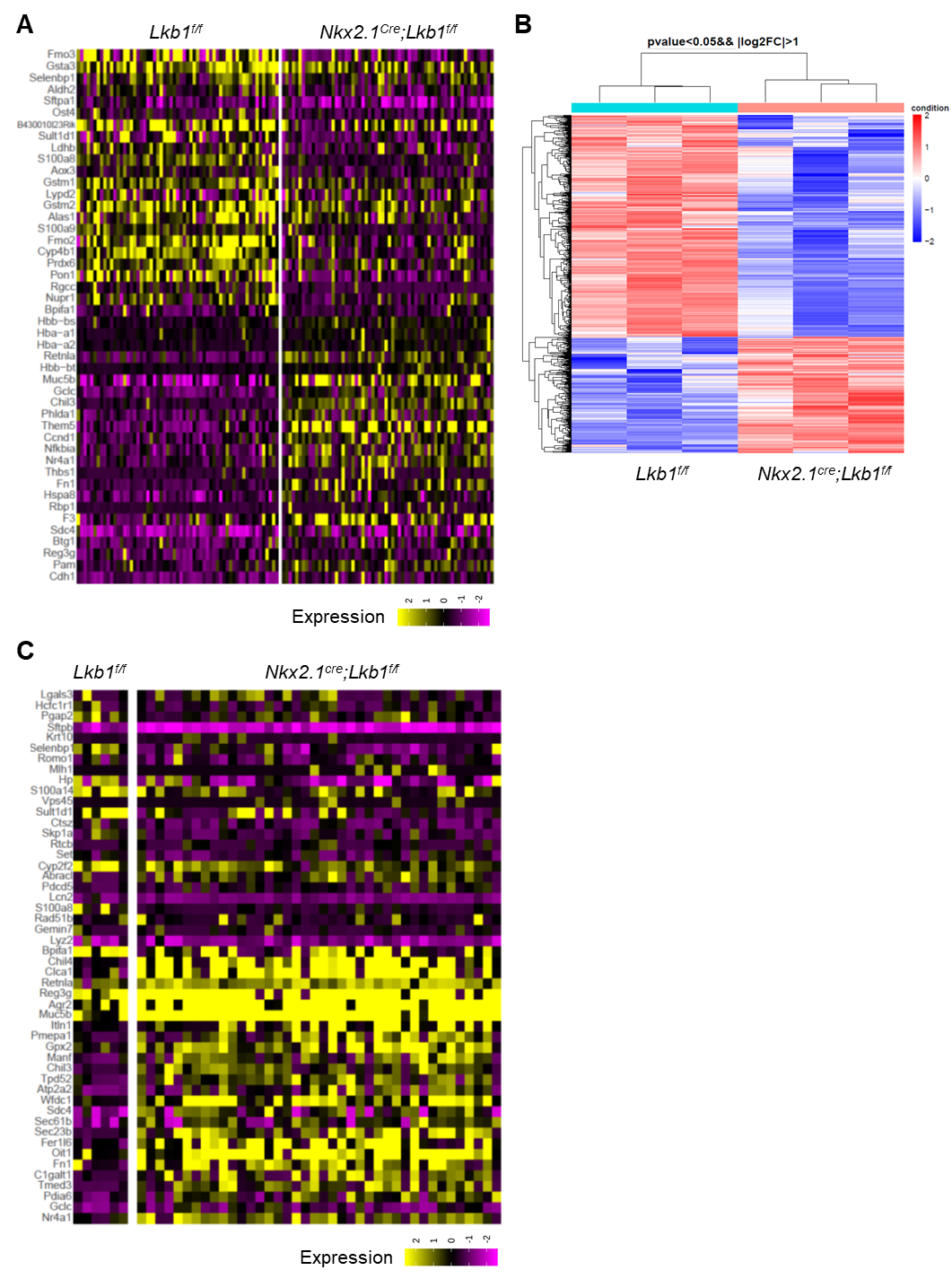
**

**Figure S7. Expression of Clca3 and RELM-α in mouse Club cells.** Immunofluorescent staining of lung sections from *Nkx2.1^Cre^;Lkb1^f/f^* mice or *Lkb1^f/f^* mice with Cyp2f2 and Clca3 or with Cyp2f2 and RELMα. Scale bar, 100 μm.

**
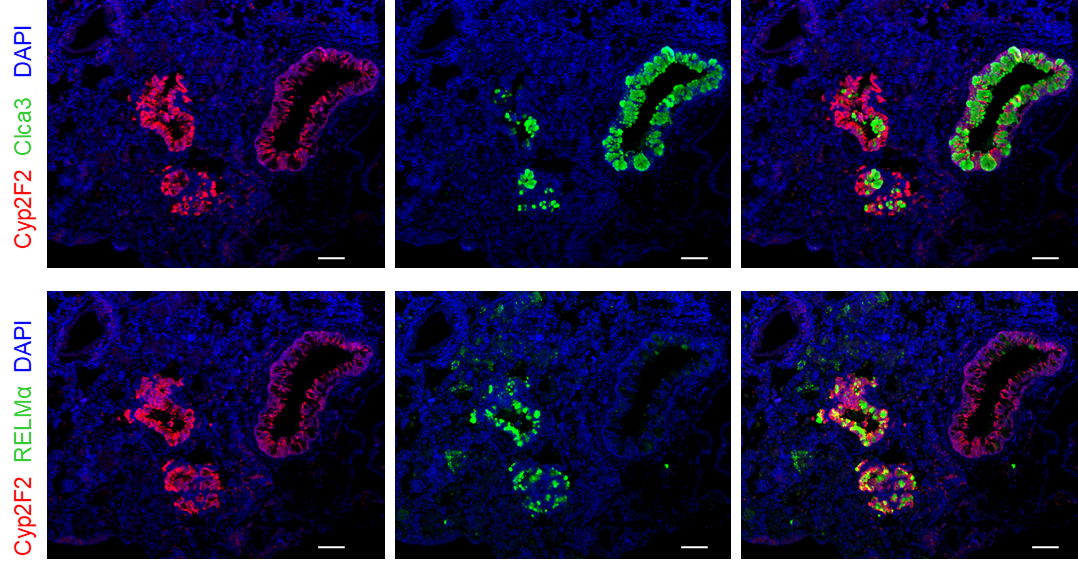
Figure S8. Organoid cultures of Club cells.** (A) Mouse Club cells were sorted by FACS. (B) Organoid cultures of club cells with or without RELM-α. (C-D) Organoid size and organoid forming ability were analyzed.


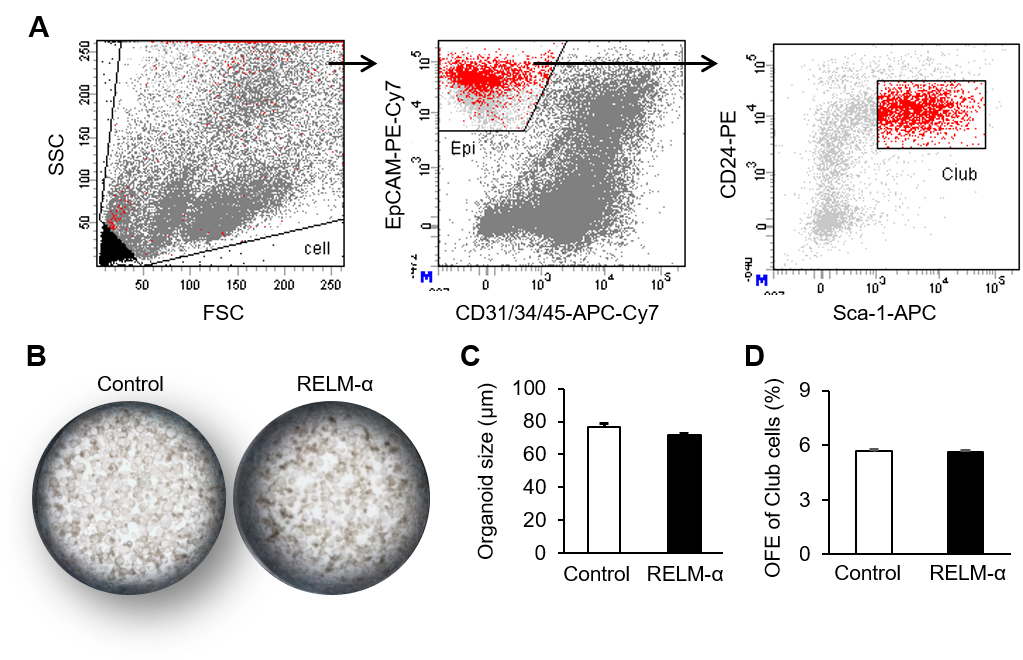


**Figure S9.** **Organoid cultures of club cells isolated from *Scgb1a1Cre;Lkb1^f/f^* mice or *Lkb1^f/f^* mice**. (A) Quantitative PCR analysis of the expression of *Lkb1* in organoid cultures of club cells from *Scgb1a1^Cre^;Lkb1^f/f^* mice or *Lkb1^f/f^* mice. (B) The expression of *Clca3* in organoid cultures of club cells from *Scgb1a1^Cre^;Lkb1^f/f^* mice or *Lkb1^f/f^* mice.

**
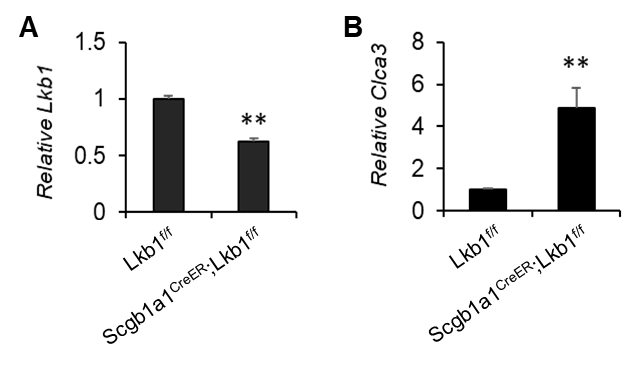
**

**Figure S10.** **Analysis of the protein-protein interaction between *Stk11* and *Retnlb* by GeneMANIA.**


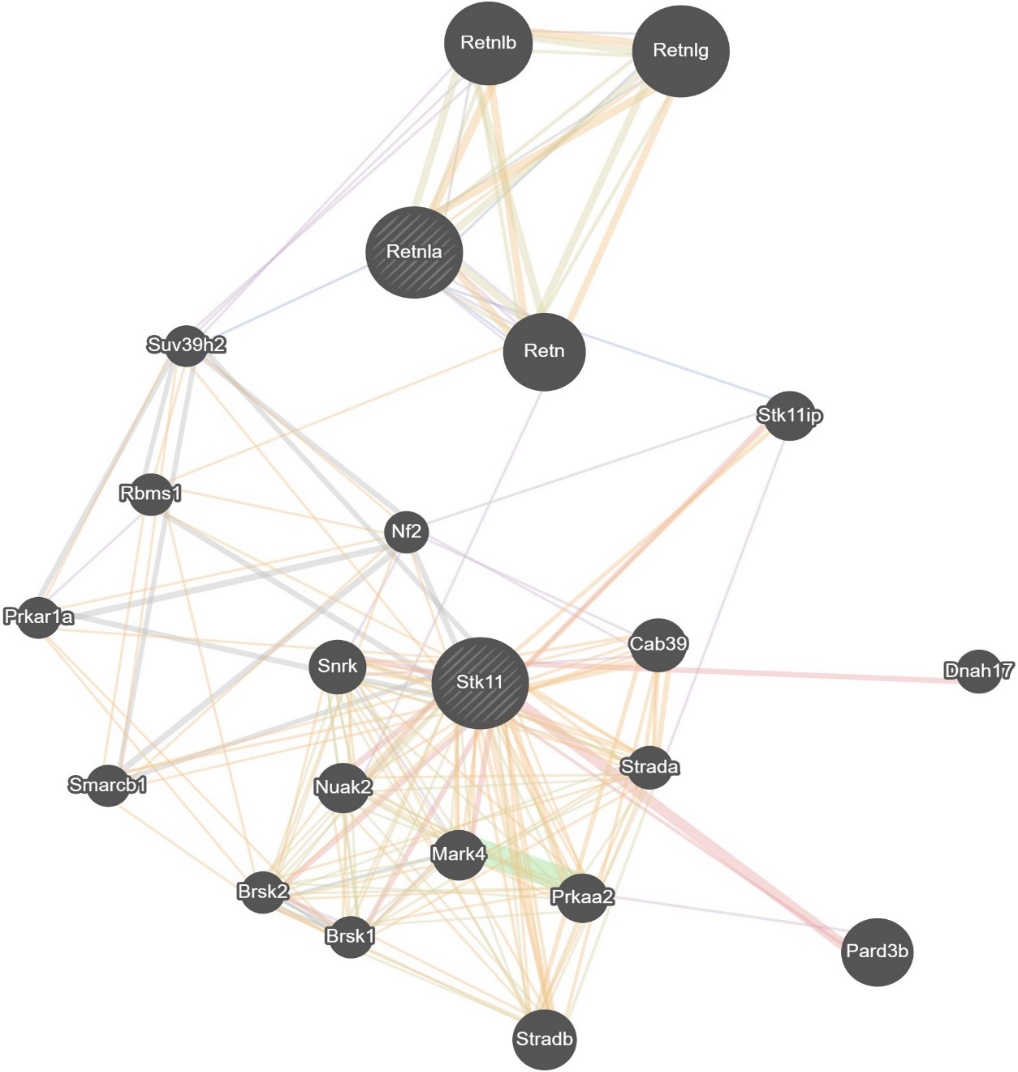


**Figure S11. Analysis of the protein-protein interaction between *Stk11* and *Retnlb* by STRING.**


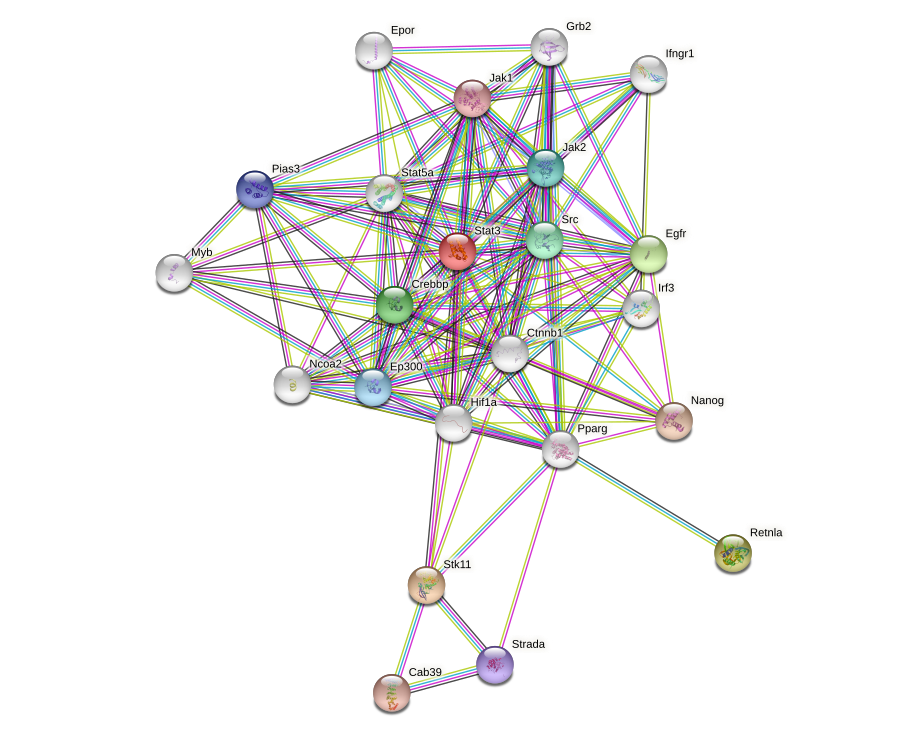


**Figure S12. YM1 expression in mouse airways**. (A) Immunofluorescent staining of lung sections from *Nkx2.1^Cre^;Lkb1^f/f^* mice versus *Lkb1^f/f^* mice with Cyp2f2 and YM1. (B) Immunofluorescent staining of lung sections from *Nkx2.1^Cre^;Lkb1^f/f^* mice versus *Lkb1^f/f^* mice with Muc5Ac and YM1.


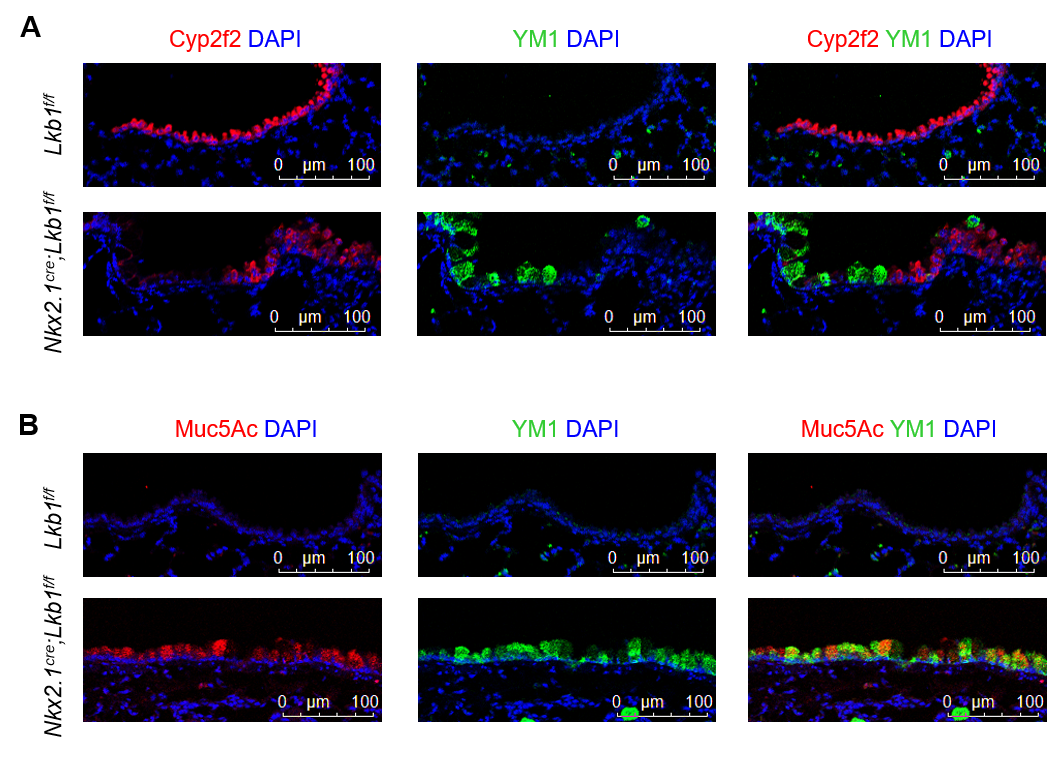


**Figure S13. Flow cytometric analysis of mouse macrophages.** Macrophages were sorted based on CD11b expression from lung tissues of C57BL/6 mice by FACS.

**
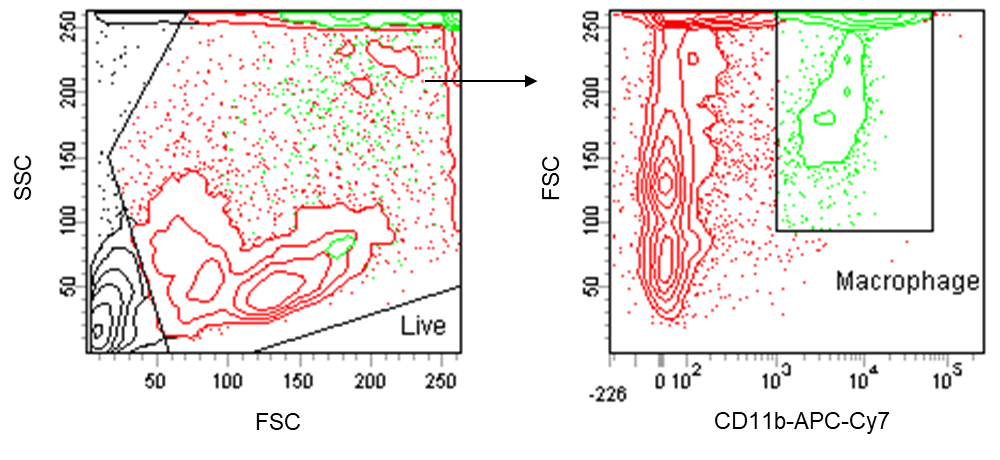
**

**Figure S14. Integrated single-cell RNA-Seq analysis of lung myeloid cells from *Nkx2.1^Cre^;Lkb1^f/f^* or *Lkb1^f/f^* mice.** The cell markers were used to identify dendritic cells, monocytes, and macrophages.


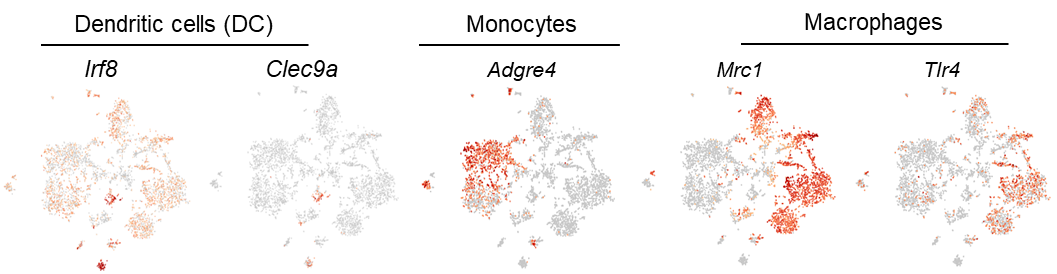

Supplement: Supplementary file 1 — Supplementary file1 (DOCX 44 kb) [file 18_2021_4044_MOESM1_ESM.docx]
