# Supplementary material for: LKB1 deficiency upregulates RELM-α to drive airway goblet cell metaplasia
Source: Cell Mol Life Sci. 2021 Dec 18;79(1):42. doi: 10.1007/s00018-021-04044-w (PMC8738459; doi:10.1007/s00018-021-04044-w)
Supplement: Supplementary file 2 — Supplementary file2 (DOCX 6075 kb) [file 18_2021_4044_MOESM2_ESM.docx]

**LKB1 deficiency upregulates RELM-α to drive airway goblet cell metaplasia**

Yu Li^1,2,3,4,#^, Qiuyang Zhang^1,2,3,4,#^, Li Li^5,#^, De Hao^1^, Peiyong Cheng^1^, Kuan Li^1,2,3,4^, Xue Li^1,2,3,4^, Jianhai Wang^1,2,3,4^, Qi Wang^2^, Zhongchao Du^2^, Hongbin Ji^6^, Huaiyong Chen^1,2,3,4,^*

^1^ Department of Basic Medicine, Haihe Hospital, Tianjin University, Tianjin, China

^2^ Key Research Laboratory for Infectious Disease Prevention for State Administration of Traditional Chinese Medicine, Tianjin Institute of Respiratory Diseases, Tianjin, China

^3^ Department of Basic Medicine, Haihe Clinical School, Tianjin Medical University, Tianjin, China

^4^ Tianjin Key Laboratory of Lung Regenerative Medicine, Tianjin, China

^5^ Department of Respiratory Medicine, Haihe Clinical School, Tianjin Medical University, Tianjin, China

^6^ State Key Laboratory of Cell Biology, CAS Center for Excellence in Molecular Cell Science, Shanghai Institute of Biochemistry and Cell Biology, Chinese Academy of Sciences, University of Chinese Academy of Sciences, Shanghai, China.

^#^ Contributed equally.

***Cellular and Molecular Life Sciences***

**Correspondence to:**

Dr. Huaiyong Chen, Department of Basic Medicine, Haihe Hospital, Tianjin University, Tianjin 300350, China, E-Mail: [huaiyong.chen@foxmail.com](mailto:huaiyong.chen@foxmail.com)

**Supplemental materials**

**Table S1.** Demographics and clinical characteristics.

| **Variables** |  | **COPD Patients** **(N=6)** | **Healthy Control** |
| --- | --- | --- | --- |
| Age-year | Mean ± SD | 71 ± 7 | 73 ± 2 |
|  | Median (IQR) | 71 (7) | 73 (3) |
|  | Range | 60 - 78 | 71 - 76 |
| Sex-no. (%) | Male | 4 (66.7) | 5 (62.5) |
|  | Female | 2 (33.3) | 3 (37.5) |
| Comorbidity-no. (%) | pneumonia | 4 (66.7) | 2 (25) |
|  | hypertension | 3 (50) | 4 (50) |
|  | diabetes | 1 (16.7) | 1 (12.5) |
|  | coronary heart disease | 3 (50) | 4 (50) |

**Table S2.** Differentially expressed genes in club cells based on scRNA-seq analysis.

| **Gene symbol** | **Fold change**  **(Increase in LKB1^-^ cells)** | ***p*** | **Gene symbol** | **Fold Change (Decrease in LKB1^-^ cells)** | ***p*** | | |
| --- | --- | --- | --- | --- | --- | --- | --- |
| Hbb-bs | 21.55 | 2.18E-06 | Aldh2 | 0.65 | | 5.13E-08 |  |
| Hba-a1 | 17.01 | 2.42E-05 | Sftpa1 | 0.64 | | 1.18E-05 |  |
| Hba-a2 | 15.61 | 5.41E-07 | Ost4 | 0.64 | | 5.57E-04 |  |
| Retnla | 9.66 | 1.23E-16 | B430010I23Rik | 0.63 | | 1.81E-05 |  |
| Hbb-bt | 9.25 | 2.14E-05 | Sult1d1 | 0.62 | | 2.50E-08 |  |
| Muc5b | 5.69 | 2.30E-13 | Ldhb | 0.62 | | 9.81E-08 |  |
| Gclc | 3.95 | 4.17E-21 | S100a8 | 0.62 | | 3.17E-13 |  |
| Chil3 | 2.70 | 2.11E-21 | Aox3 | 0.61 | | 5.58E-09 |  |
| Phlda1 | 2.65 | 3.25E-09 | Gstm1 | 0.60 | | 1.34E-10 |  |
| Them5 | 2.55 | 1.69E-17 | Lypd2 | 0.60 | | 7.11E-03 |  |
| Ccnd1 | 2.33 | 1.16E-09 | Gstm2 | 0.58 | | 6.91E-09 |  |
| Nfkbia | 2.32 | 5.01E-10 | Alas1 | 0.57 | | 8.09E-05 |  |
| Nr4a1 | 2.24 | 2.98E-09 | S100a9 | 0.57 | | 2.39E-08 |  |
| Thbs1 | 2.17 | 2.58E-03 | Fmo2 | 0.52 | | 2.87E-11 |  |
| Fn1 | 2.17 | 2.28E-14 | Cyp4b1 | 0.51 | | 1.30E-13 |  |
| Hspa8 | 2.12 | 2.29E-12 | Prdx6 | 0.49 | | 2.47E-12 |  |
| Rbp1 | 2.04 | 4.07E-05 | Pon1 | 0.49 | | 4.21E-12 |  |
| F3 | 2.03 | 3.22E-05 | Rgcc | 0.49 | | 6.12E-12 |  |
| Sdc4 | 2.00 | 2.30E-07 | Nupr1 | 0.43 | | 1.23E-13 |  |
| Btg1 | 1.97 | 3.61E-06 | Bpifa1 | 0.34 | | 4.82E-03 |  |

**Table S3.** Bulk RNA-seq analysis of lung epithelial cells.

| **Gene symbol** | **Fold Change**  **(Increase in LKB1^-^ cells)** | ***p*** | **Gene symbol** | **Fold Change (Decrease in LKB1^-^ cells)** | ***p*** |
| --- | --- | --- | --- | --- | --- |
| 5730507C01Rik | Inf | 9.7E-07 | Fam47e | 0.250 | 1.4E-04 |
| A2m | Inf | 8.2E-03 | Parm1 | 0.249 | 1.8E-16 |
| Acvr1c | Inf | 5.5E-03 | Fam216b | 0.249 | 4.6E-12 |
| Adipoq | Inf | 2.9E-02 | Caps2 | 0.249 | 4.0E-04 |
| Alx4 | Inf | 4.7E-03 | Gm28729 | 0.248 | 3.4E-12 |
| Cyp4a12a | Inf | 2.5E-04 | Cabp2 | 0.247 | 1.2E-02 |
| Foxi2 | Inf | 1.6E-05 | Gm3636 | 0.243 | 1.5E-03 |
| Gm14461 | Inf | 1.1E-02 | Prr18 | 0.243 | 1.9E-06 |
| Gm4131 | Inf | 1.3E-02 | Stmnd1 | 0.242 | 1.2E-12 |
| Lipn | Inf | 1.8E-03 | Bbox1 | 0.241 | 2.0E-02 |
| Lrp1b | Inf | 1.6E-02 | Ldlrad1 | 0.241 | 3.2E-16 |
| Mrap | Inf | 1.4E-02 | A830018L16Rik | 0.241 | 1.8E-05 |
| Msx3 | Inf | 8.9E-03 | Hhatl | 0.241 | 3.5E-03 |
| Npy | Inf | 1.5E-02 | Cfap100 | 0.240 | 5.3E-11 |
| Oosp1 | Inf | 1.7E-05 | Krt79 | 0.239 | 6.1E-03 |
| Prss46 | Inf | 8.8E-03 | Lpar3 | 0.238 | 4.0E-25 |
| Ptgdr | Inf | 1.2E-02 | Hs3st6 | 0.237 | 2.2E-07 |
| Sprr2a1 | Inf | 2.9E-02 | Pih1h3b | 0.236 | 4.4E-03 |
| Tff1 | Inf | 2.3E-02 | Cox6a2 | 0.235 | 1.2E-07 |
| Rnase2a | 492.37 | 2.6E-03 | Odf3b | 0.235 | 8.5E-08 |
| Pax7 | 169.32 | 1.1E-73 | 1700024G13Rik | 0.230 | 1.6E-09 |
| Fbp1 | 149.50 | 1.5E-05 | Ccdc121 | 0.230 | 1.0E-06 |
| Chil4 | 136.83 | 3.1E-02 | Sec14l5 | 0.229 | 1.1E-06 |
| Arg1 | 39.20 | 6.6E-03 | 4932443I19Rik | 0.229 | 4.6E-04 |
| Lancl3 | 32.30 | 9.6E-15 | Tmem232 | 0.229 | 1.5E-05 |
| Kcnq2 | 29.00 | 1.2E-03 | Rsph4a | 0.229 | 1.8E-18 |
| Pde10a | 28.56 | 4.7E-09 | Morn3 | 0.226 | 6.2E-09 |
| Car6 | 27.70 | 1.5E-06 | Grm4 | 0.226 | 1.1E-03 |
| Gpr176 | 27.05 | 9.0E-04 | Adcyap1r1 | 0.226 | 7.2E-09 |
| Mmp12 | 26.27 | 4.9E-02 | Drc1 | 0.225 | 2.2E-14 |
| Corin | 25.14 | 1.2E-02 | Fam159a | 0.224 | 4.3E-02 |
| Prkag3 | 25.05 | 3.4E-03 | Cyp4b1 | 0.221 | 1.5E-39 |
| Rnase2b | 24.55 | 1.5E-02 | Ggt6 | 0.219 | 5.0E-05 |
| Lemd1 | 20.40 | 6.5E-03 | Cdh15 | 0.218 | 2.5E-02 |
| Kcnc4 | 20.35 | 8.5E-12 | Mlana | 0.218 | 8.8E-03 |
| Gtsf1 | 19.49 | 4.8E-02 | Col8a2 | 0.217 | 7.2E-12 |
| Rgs4 | 18.82 | 1.1E-06 | Rp1 | 0.215 | 1.1E-09 |
| Spp1 | 17.79 | 7.8E-30 | Fmo6 | 0.215 | 3.8E-04 |
| Ceacam15 | 17.00 | 2.8E-02 | Gm11992 | 0.215 | 3.1E-08 |
| Lhx2 | 16.24 | 7.6E-03 | Ankef1 | 0.214 | 5.6E-03 |
| Cap2 | 15.15 | 1.6E-22 | Atp13a4 | 0.214 | 1.7E-13 |
| Nkx2-2 | 14.71 | 2.7E-19 | Serpina10 | 0.214 | 1.6E-03 |
| Mlip | 14.41 | 3.6E-02 | Aqp4 | 0.214 | 4.7E-20 |
| Tph2 | 13.70 | 4.6E-02 | Camk2b | 0.213 | 2.6E-05 |
| Cdh10 | 13.64 | 4.6E-02 | Sntn | 0.211 | 5.1E-14 |
| Tmem26 | 12.17 | 2.5E-02 | Iqca | 0.211 | 4.2E-18 |
| Retnla | 12.13 | 2.3E-02 | Ccdc180 | 0.211 | 3.4E-20 |
| Prr16 | 11.85 | 9.4E-05 | Ppm1n | 0.211 | 3.5E-02 |
| Sox9 | 11.77 | 2.1E-14 | Tymp | 0.210 | 5.0E-06 |
| Rbm24 | 11.55 | 4.2E-22 | Fam81a | 0.209 | 3.5E-16 |
| Slc26a4 | 11.51 | 1.6E-02 | Agtr1a | 0.205 | 3.9E-05 |
| H2-M2 | 11.08 | 2.9E-02 | Slurp2 | 0.204 | 9.5E-03 |
| Ceacam19 | 10.63 | 2.3E-02 | Cpn2 | 0.201 | 3.7E-05 |
| Grid1 | 10.59 | 2.2E-03 | Rptn | 0.200 | 1.9E-06 |
| Pdzrn4 | 10.26 | 1.1E-03 | Slc19a3 | 0.199 | 3.4E-07 |
| Mest | 10.22 | 2.8E-33 | Rd3 | 0.199 | 4.6E-08 |
| Igf1 | 10.17 | 5.4E-03 | Ces1b | 0.197 | 1.8E-04 |
| Rbp1 | 9.87 | 2.8E-03 | Dcdc5 | 0.196 | 1.3E-02 |
| Ttr | 9.26 | 1.5E-03 | Cfap161 | 0.195 | 3.5E-10 |
| Kcnf1 | 9.17 | 4.2E-14 | Gm281 | 0.190 | 2.0E-10 |
| Ccdc154 | 9.07 | 2.4E-04 | Cyp4a12b | 0.185 | 2.0E-09 |
| Baiap2l2 | 8.62 | 5.4E-09 | Fmo3 | 0.185 | 9.9E-17 |
| Tceal3 | 8.43 | 1.3E-25 | Ttc34 | 0.184 | 5.4E-14 |
| Pde1c | 8.36 | 1.4E-12 | Cfap221 | 0.182 | 3.3E-08 |
| Tnni3k | 8.22 | 3.5E-05 | Asgr1 | 0.169 | 2.7E-08 |
| Slc22a19 | 8.20 | 5.9E-04 | Slc47a2 | 0.154 | 5.0E-02 |
| Gm4951 | 8.13 | 7.9E-03 | Egfem1 | 0.149 | 1.6E-06 |
| Kng2 | 8.12 | 3.1E-04 | Iqch | 0.149 | 5.6E-04 |
| Ptgir | 7.99 | 1.0E-03 | Spink5 | 0.149 | 3.6E-09 |
| Gm39701 | 7.96 | 3.5E-05 | Pon1 | 0.147 | 3.3E-58 |
| Cspg4 | 7.86 | 9.7E-05 | Htr1b | 0.144 | 4.4E-04 |
| Slc22a1 | 7.63 | 4.2E-02 | Ephx3 | 0.144 | 3.9E-02 |
| Jchain | 7.59 | 2.0E-02 | Hes2 | 0.140 | 6.7E-19 |
| Hecw1 | 7.59 | 2.8E-02 | Ccdc42 | 0.132 | 9.0E-03 |
| Cpa2 | 7.37 | 2.7E-02 | Ces1f | 0.125 | 1.4E-12 |
| Sorbs2 | 7.32 | 1.3E-22 | Abra | 0.124 | 1.5E-03 |
| Lrrc39 | 7.27 | 6.6E-05 | Bpifa1 | 0.119 | 4.5E-14 |
| Fxyd4 | 7.15 | 2.4E-02 | Adamts20 | 0.118 | 2.7E-03 |
| Cdkn2a | 7.10 | 1.2E-02 | Nos2 | 0.114 | 2.0E-30 |
| Rimbp2 | 7.06 | 2.4E-12 | Tmem212 | 0.110 | 1.4E-05 |
| Gli2 | 6.68 | 6.7E-09 | Ces1g | 0.103 | 3.6E-04 |
| Inhba | 6.68 | 1.0E-03 | Fga | 0.097 | 1.2E-02 |
| Sbk2 | 6.64 | 2.7E-06 | Shisa6 | 0.093 | 2.2E-11 |
| Ahsg | 6.57 | 1.3E-03 | Nrn1 | 0.093 | 7.7E-19 |
| Phex | 6.35 | 6.2E-07 | Sult1d1 | 0.085 | 2.1E-25 |
| Ces2c | 6.17 | 6.7E-03 | Jakmip2 | 0.081 | 7.8E-03 |
| Cers3 | 6.13 | 2.6E-06 | Apobec4 | 0.068 | 4.8E-03 |
| Fstl4 | 6.11 | 9.9E-08 | Krt86 | 0.061 | 2.8E-02 |
| Slc24a5 | 5.99 | 6.0E-03 | Avpr1a | 0.060 | 1.3E-13 |
| Morc1 | 5.96 | 5.4E-06 | Mkrn2os | 0.057 | 2.1E-02 |
| Sbspon | 5.94 | 6.0E-03 | Cilp2 | 0.050 | 1.2E-02 |
| Daam2 | 5.84 | 8.8E-19 | Sult2a1 | 0.049 | 1.2E-02 |
| Gp2 | 5.71 | 4.0E-02 | Uty | 0.003 | 4.4E-02 |
| Tnc | 5.63 | 5.9E-10 | Kdm5d | 0.002 | 3.1E-02 |
| Olfr111 | 5.56 | 3.7E-02 | Eif2s3y | 0.001 | 2.2E-02 |
| LOC108168067 | 5.50 | 1.3E-02 | Ddx3y | 0.000 | 1.8E-02 |
| Ctsk | 5.41 | 3.4E-04 | En2 | 0.000 | 4.4E-02 |
| Ch25h | 5.41 | 7.8E-20 | Spats1 | 0.000 | 2.4E-02 |
| Stra6l | 5.36 | 5.8E-05 | Wfdc13 | 0.000 | 6.5E-03 |
| Cntn1 | 5.35 | 7.4E-04 |  |  |  |
| Rimkla | 5.31 | 1.3E-05 |  |  |  |
| Rag1 | 5.20 | 7.4E-03 |  |  |  |
| Gypa | 5.16 | 1.6E-02 |  |  |  |
| Myh6 | 5.06 | 2.0E-03 |  |  |  |
| Pak3 | 5.05 | 5.4E-03 |  |  |  |
| Gfra4 | 5.05 | 5.3E-04 |  |  |  |
| Adamts15 | 5.04 | 4.3E-06 |  |  |  |
| Fabp4 | 4.92 | 3.5E-05 |  |  |  |
| Chil3 | 4.92 | 2.2E-03 |  |  |  |
| Rhpn1 | 4.87 | 1.7E-03 |  |  |  |

**Table S4.** Differentially expressed genes in goblet cells based on scRNA-seq analysis.

| **Gene symbol** | **Fold Change**  **(Increase in LKB1^-^ cells)** | ***p*** | **Gene symbol** | **Fold Change (Decrease in LKB1^-^ cells)** | | ***p*** |
| --- | --- | --- | --- | --- | --- | --- |
| Chil4 | 176.97 | 2.3E-06 | Lgals3 | 0.47 | 1.5E-02 | |
| Clca1 | 79.23 | 1.8E-05 | Hcfc1r1 | 0.46 | 2.0E-02 | |
| Retnla | 10.70 | 1.9E-07 | Pgap2 | 0.46 | 1.4E-02 | |
| Reg3g | 7.07 | 7.2E-03 | Sftpb | 0.43 | 1.0E-02 | |
| Agr2 | 5.28 | 3.9E-03 | Krt10 | 0.43 | 1.2E-04 | |
| Muc5b | 5.03 | 5.4E-05 | Selenbp1 | 0.43 | 1.0E-04 | |
| Itln1 | 3.55 | 4.0E-03 | Romo1 | 0.43 | 6.4E-03 | |
| Pmepa1 | 3.33 | 5.7E-03 | Mlh1 | 0.42 | 5.6E-04 | |
| Gpx2 | 3.13 | 9.8E-03 | Hp | 0.42 | 1.9E-02 | |
| Manf | 3.09 | 1.6E-03 | S100a14 | 0.42 | 8.1E-03 | |
| Chil3 | 2.82 | 4.7E-05 | Vps45 | 0.42 | 1.1E-03 | |
| Tpd52 | 2.80 | 4.3E-03 | Sult1d1 | 0.42 | 2.8E-03 | |
| Atp2a2 | 2.77 | 3.3E-03 | Ctsz | 0.42 | 7.5E-03 | |
| Wfdc1 | 2.75 | 2.5E-03 | Skp1a | 0.41 | 2.3E-03 | |
| Sdc4 | 2.74 | 3.7E-03 | Rtcb | 0.40 | 4.2E-02 | |
| Sec61b | 2.73 | 3.0E-03 | Set | 0.40 | 1.8E-02 | |
| Sec23b | 2.70 | 6.0E-04 | Cyp2f2 | 0.40 | 8.4E-03 | |
| Fer1l6 | 2.64 | 2.7E-03 | Abracl | 0.39 | 1.8E-02 | |
| Oit1 | 2.62 | 4.2E-02 | Pdcd5 | 0.38 | 1.9E-02 | |
| Fn1 | 2.56 | 1.4E-03 | Lcn2 | 0.34 | 2.3E-02 | |
| C1galt1 | 2.51 | 1.0E-02 | S100a8 | 0.33 | 7.8E-04 | |
| Tmed3 | 2.50 | 2.9E-04 | Rad51b | 0.30 | 4.2E-03 | |
| Pdia6 | 2.50 | 6.7E-03 | Gemin7 | 0.29 | 8.9E-03 | |
| Gclc | 2.48 | 2.9E-02 | Lyz2 | 0.18 | 2.0E-03 | |
| Nr4a1 | 2.47 | 2.0E-02 | Bpifa1 | 0.06 | 1.9E-04 | |
